# CRX is an intrinsic suppressor of epithelial‒mesenchymal transition in retinal pigment epithelial cells: a promising therapeutic avenue for subretinal fibrosis

**DOI:** 10.1038/s41419-025-08352-y

**Published:** 2025-12-31

**Authors:** Dongli Li, Qingjian Ou, Furong Gao, Xi Wang, Lilin Zhu, Ye Zhou, Jing-Ying Xu, Caixia Jin, Juan Wang, Jieping Zhang, Jiao Li, Yanlong Bi, Lixia Lu, Guo-Tong Xu, Haibin Tian

**Affiliations:** 1 Department of Ophthalmology of Tongji Hospital and Laboratory of Clinical and Visual Sciences of Tongji Eye Institute, School of Medicine, Tongji University, Shanghai, China; 2 Department of Ophthalmology, Suzhou Municipal Hospital & the Affiliated Suzhou Hospital of Nanjing Medical University, Nanjing Medical University, Suzhou, China; 3 Department of Pharmacology, School of Medicine, Tongji University, Shanghai, China; 4 Teaching Laboratory Center, School of Medicine, Tongji University, Shanghai, China

**Keywords:** HIPPO signalling, Translational research

## Abstract

The epithelial-mesenchymal transition (EMT) of retinal pigment epithelial (RPE) cells is one of the significant pathogenic mechanisms for the formation of subretinal fibrosis in age-related macular degeneration (AMD). Multiple signaling pathways that promote EMT have been well described, yet the endogenous signaling pathways that inhibit EMT within RPE cells remain largely elusive. In this study, we confirmed the expression of CRX in human RPE cells and human embryonic stem cell-derived RPE (ESC-RPE) cells. By employing sub-culture to disrupt intercellular connections and thereby inhibit the Hippo signaling pathway, combined with TGF-β1 treatment in vitro to mimic the microenvironment for the formation of subretinal fibrosis, it was revealed that Hippo/YAP1 and TGF-β1 synergistically promoted the nuclear translocation of β-catenin, and the latter bound to TCF7 to inhibit the expression of CRX. Overexpression of CRX was capable of suppressing the occurrence of EMT in ESC-RPE cells. CRX exerted its inhibitory effect on EMT partly by upregulating the expression of PPP2R2B. In the laser-induced choroidal neovascularization mouse model, the nuclear translocation of CRX took place in RPE cells, and overexpression of CRX played an inhibitory role in the formation of subretinal fibrosis. This study has identified CRX as an endogenous signaling molecule that inhibits EMT in RPE cells and has provided a new research target and treatment strategy for the treatment of wet AMD and the inhibition of subretinal fibrosis formation.

## Introduction

Macular neovascularization (MNV) is an important cause of blindness in wet age-related macular degeneration (AMD) patients. There are three main types of MNVs: Type 1 MNV refers to choroidal blood vessels expanding below the RPE, type 2 MNV is characterized by proliferating choroidal blood vessels breaking through Bruch’s membrane and the RPE monolayer to spread in the subretinal space, and type 3 MNV originates from the retinal vasculature and progresses posteriorly into the subretinal space [1]. The risk of eyes with wet AMD developing subretinal fibrosis (SF) despite ongoing anti-VEGF therapy is reported to be 45% by two years [2] and 41% by ten years [3]. Among the three types, type 2 MNV is associated with more SF than other MNV types [4], which is a major risk factor for poor visual outcomes after treatment [5]. RPE cells undergo epithelial-mesenchymal transition (EMT) to transform into myofibroblasts is the most important contribution to the formation of SF [6]. The proportion of RPE cells undergoing EMT in this fibrotic tissue has been reported to reach 40% [7]. Therefore, inhibiting EMT in RPE cells has always been an attractive research topic in the treatment of SF [8, 9]. Currently, multiple EMT-related signaling pathways have been reported, such as the TGF-β signaling pathway, BMP signaling pathway, Wnt/β-catenin signaling pathway, and Hippo/YAP signaling pathway, and the use of inhibitors to inhibit these signaling pathways can inhibit EMT in RPE cells [10, 11]. However, whether there are endogenous signaling pathways that inhibit EMT in RPE cells, especially when EMT occurs, whether endogenous signaling pathways can be activated to inhibit EMT remains unclear.

When we cultured embryonic stem cell-derived RPE (ESC-RPE) cells and induced pluripotent stem cell-derived RPE (iPSC-RPE) cells, the cells can be passaged multiple times in vitro at a 50% ratio while still maintaining an epithelial state [12, 13]. The process of cell passage is actually a process of EMT followed by mesenchymal-epithelial transition (MET). This finding indicates the existence of endogenous signaling pathways in the cells that inhibit EMT or promote MET, ensuring that the cells can return to the epithelial state. We previously successfully obtained iRPE cells via the use of key transcription factors, which have the function of resisting EMT, and determined that the key transcription factor cone‒rod homeobox containing gene (CRX) plays the most important role in resisting EMT [12, 14]. CRX is expressed mainly in cone cells and rod cells and is an important transcription factor that regulates the differentiation of photoreceptor cells [15] and the manifestation of its expression in RPE cells is controversial [16–19], and its function in RPE cells is not clear. In this article, we aim to ascertain the expression of endogenous CRX in RPE cells and explore whether CRX exerts an inhibitory effect on the EMT in RPE cells. Additionally, we will elucidate the upstream and downstream regulatory signaling pathways of CRX. Moreover, a laser-induced choroidal neovascularization (CNV) mouse model will be used to verify that CRX inhibits EMT in RPE cells, consequently impeding the formation of SF.

## Results

### CRX is expressed in RPE cells

**Fig. 1:**
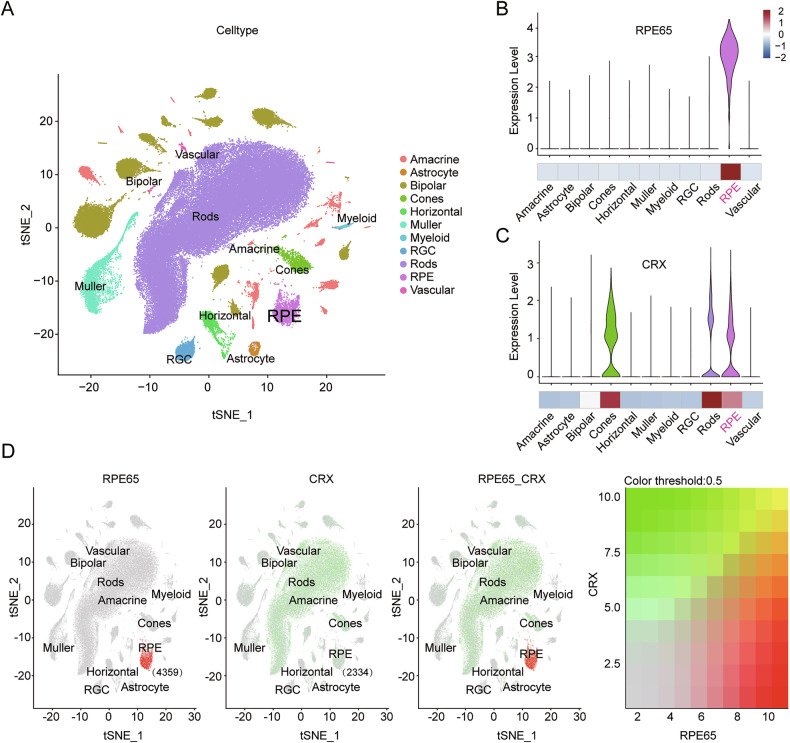
The expression of *CRX* in human RPE cells is determined by the single-nuclei RNA-seq. **A** Uniform manifold approximation and projection (UMAP) dimensionality reduction plot showed the profiled cells including amacrine, astrocyte, bipolar, cone, horizontal, muller, myeloid, RGC, rod, RPE, vascular cells. **B**, **C** The violin plots of *RPE65* and *CRX* expression in all cell types. **D** The co-expressions of *RPE65* and *CRX*. Red indicates the expression of *RPE65* (4359 RPE cells), green indicates the expression of *CRX* (2334 RPE cells); yellow indicates the co-expressions of *RPE65* and *CRX*, the ratio of CRX + RPE cells was 53.54%.

CRX is a crucial transcription factor that regulates the development of photoreceptor cells [15]. Nevertheless, the manifestation of its expression within RPE cells is controversial [16–19]. To determine whether CRX is expressed in RPE cells, we analyzed published single-cell sequencing data based on the identified cell markers [19]. Various types of cells were observed from the retina and RPE/choroid, including amacrine, astrocyte, bipolar, cone, horizontal, muller, myeloid, RGC, rod, RPE, and vascular cells (Fig. 1A). Our observations revealed that *RPE65* was exclusively expressed in RPE cells (Fig. 1B). Moreover, CRX is highly expressed in rod and cone cells, while detectable levels in RPE cells suggest a potential regulatory role in RPE biology or its interaction with photoreceptors, albeit less dominant than its established functions in photoreceptors. (Fig. 1C). Notably, a total of 4359 RPE cells (expressing *RPE65*) were counted. Among them, 2334 RPE cells were found to express *CRX* (Fig. 1D). Consequently, the proportion of RPE cells expressing *CRX* reached 53.54%. Western blotting results showed that CRX was expressed in both ESC-RPE cells and iPSC-RPE cells (Supplementary Fig. 1A). Furthermore, an analysis of previously published gene microarray data [20] indicated that the expression of *CRX* could be detected in RPE-choroid tissues (Supplementary Fig. 1B). Collectively, these results demonstrate that there is a certain amount of *CRX* expression in RPE cells.

### The expression level and the nucleus translocation of CRX demonstrate dynamic alterations during the sub-culturing process of RPE cells

**Fig. 2:**
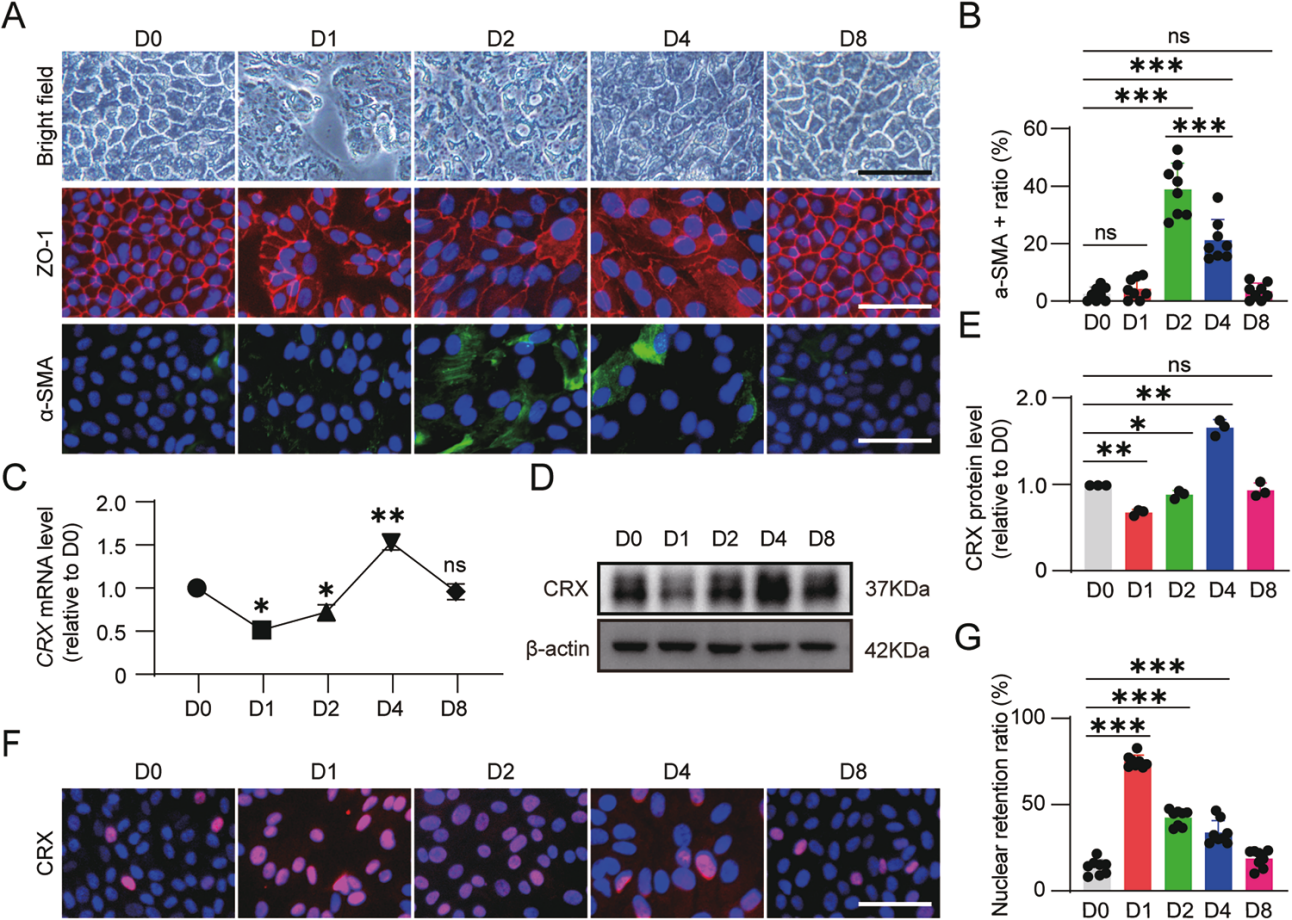
The expression level of CRX and its kinetic changes of nuclear retention during the sub-culturing of ESC-RPE cells. **A** Changes in cell morphology, and the expressions of ZO-1 and α-SMA at different time points during the cell sub-culturing process. **B** Quantitative calculation of the percentage of α-SMA^+^ cells (*n* = 8). Detection of the mRNA and protein expression levels of CRX at different time points by (**C**) qRT-PCR, (**D**) Western blotting, and (**E**) quantitative analysis (*n* = 3). **F**, **G** Nuclear retention ratio of CRX at different time points during the cell sub-culturing process (*n* = 8). Scale bar = 50 μm. Data are presented as mean ± SD. Statistical significance was defined as follows: *p < 0.05, **p < 0.01, ***p < 0.001; ns, not significant (using one-way ANOVA and post hoc Bonferroni’s test).

In the context of Type 2 MNV, the choroidal vasculature penetrates the Bruch’s membrane and the RPE cell layer [1], that invariably results in the disruption of tight junctions among RPE cells. Cell sub-culturing, which also disrupts intercellular connections, can mimic this process. Our previous investigations have demonstrated that RPE cells are able to be sub-cultured at a 50% ratio for at least five passages [12, 13]. When cells are sub-cultured, their tight junctions became discontinuously and subsequently re-established (Fig. 2A). The expression level of α-SMA exhibits a gradual increase of sub-culture, followed by a gradual decline to the initial level when the cells demonstrated polygonal epithelial morphology again (Fig. 2A, B). This phenomenon corroborates that the cells first experience the EMT process and then the MET process.

We further analyzed the expression of CRX and observed that the expression level of CRX decreased on the first day of sub-culture. Subsequently, concomitant with the formation of tight junctions (as indicated by ZO-1 expression in Fig. 2A), the expression of CRX gradually augmented, peaking on the fourth day, and then decreased subsequent to the initial level (Fig. 2C–E). Additionally, we discovered that when the cells formed tight junctions and assumed a polygonal morphology, the proportion of cells with positive CRX nuclear staining was less than 15% (Fig. 2F, G). On the first day of cell sub-culturing, CRX translocated into the nucleus, the nuclear retention ratio was over 75% (Fig. 2F, G). Thereafter, the nuclear retention ratio of CRX was gradually reduced (Fig. 2F, G). Collectively, these findings suggest that the expression and nuclear shuttling of CRX are intricately associated with the EMT and MET processes of RPE cells.

### β-Catenin/TCF7 mediates the inhibitory effect of YAP1 on CRX expression

**Fig. 3:**
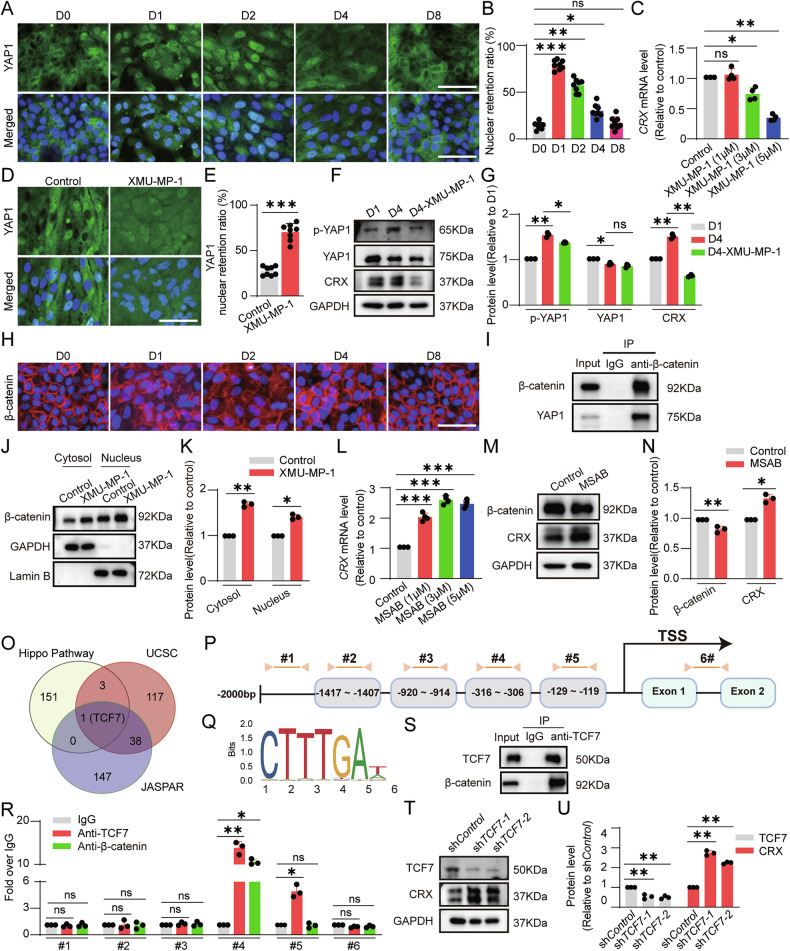
β-Catenin/TCF7 mediates the inhibitory effect of YAP1 on CRX expression in ESC-RPE cells. **A, B** The images of nuclear retention of YAP1 at different time points during sub-culturing of ESC-RPE cells and quantitative analysis (*n* = 8). **C** The CRX expression level in ESC-RPE cells after being treated with the Hippo pathway inhibitor XMU-MP-1 for four days was detected by qRT-PCR (*n* = 3). **D** After treatment with 5 μM XMU-MP-1 for four days, the nuclear retention of YAP1 was detected by immunostaining. **E** The quantitative analysis of YAP1 nuclear retention ratio (*n* = 8). **F**, **G** The expression levels of p-YAP1, YAP1, and CRX in ESC-RPE cells after being treated with 5 μM XMU-MP-1 for four days were determined and quantified by Western blotting (n = 3). **H** The cellular localization of β-catenin at different time points during sub-culturing was demonstrated by immunostaining. **I** Co-IP analysis of the interaction between β-catenin and YAP1. **J**, **K** The protein level of β-catenin in the nucleus and cytoplasm of cells after being treated with 5 μM XMU-MP-1 for four days was determined and quantified by Western blotting (n = 3). **L** The CRX expression level in ESC-RPE cells after being treated with β-catenin inhibitor MSAB for four days was detected by qRT-PCR (n = 3). **M**, **N** The expression levels of β-catenin and CRX in ESC-RPE cells after being treated with 3 μM MSAB for four days were determined and quantified by Western blotting (n = 3). **O** Venn analysis was conducted on Hippo signaling pathway-related proteins (https://www.genecards.org/), transcription factors predicted to bind to the CRX promoter in the UCSC database (https://genome.ucsc.edu/) and the JAPSAR database (https://jaspar.elixir.no/), the candidate protein TCF7 was screened out. **P** The regions of the CRX gene paired with qRT-PCR primers (#1–5: promoter, #6: intron). **Q** The matrices of β-catenin and TCF7 proteins binding to the CRX gene promoter predicted by the JASPAR database. **R** The binding sites of β-catenin and TCF7 to the *CRX* promoter were verified by ChIP-qRT-PCR (*n* = 3). **S** CoIP analysis of the interaction between TCF7 and β-catenin. **T-U** TCF7 was knocked down in ESC-RPE cells, and the expression levels of TCF7 and CRX were determined and quantified by Western blotting (*n* = 3). Scale bar = 50 μm. Data are presented as mean ± SD. Statistical significance was defined as follows: *p < 0.05, **p < 0.01, ***p < 0.001; ns not significant (using unpaired two-sided t-tests in **E**, **K**, and **N** and one-way ANOVA and post hoc Bonferroni’s test in the others).

Cell-cell junction disruption rapidly inhibits the Hippo signaling pathway, reducing the level of the phosphorylated transcriptional coactivator YAP1 [21]. The latter translocates into the nucleus and induces EMT in RPE cells [22, 23]. We found that after cell sub-culturing, YAP1 also exhibits kinetic changes in nuclear entry and exit. One day after sub-culturing, a large amount of YAP1 entered the nucleus, after which the amount of YAP1 in the nucleus gradually decreased (Fig. 3A, B). We further conducted a scratch assay and found that the cells at the edge of the scratch lost contact inhibition and underwent migration (Supplementary Fig. 2A). The cells migrating in the front were completely dispersed and named disseminated cells, while the cells migrating slowly behind were named slow-migrating cells (Supplementary Fig. 2A). Two days after the scratch, a large number of disseminated cells expressed CRX in their cell nuclei, along with a small number of α-SMA-positive cells (Supplementary Fig. 2B). Seven days later, the disseminated cells completely lost their epithelial-like tight junctions, taking on a dedifferentiated fibroblast-like appearance (Supplementary Fig. 2C). In addition, both slow-migrating cells and disseminated cells expressed α-SMA, and almost no disseminated cells demonstrated nuclear retention of CRX (Supplementary Fig. 2B, D). Meanwhile, YAP1 was still present in the cell nuclei of the disseminated cells (Supplementary Fig. 2D). These results demonstrated that in slow-migrating cells, both CRX and α-SMA were expressed, and these cells corresponded to the state of cells 1–4 days after passage. In contrast, in disseminated cells, due to the cells being in a highly migratory state, intercellular tight junctions could not be formed, the Hippo signaling pathway was continuously inhibited, CRX expression was reduced, and CRX was absent in the nucleus, leading to the complete EMT of the cells. These data suggest that inhibition of the Hippo signaling pathway leads to subsequent activation of YAP1, which may inhibit the expression of CRX and thereby lead to the occurrence of EMT in RPE cells. In order to address that the Hippo signaling pathway regulates CRX expression, we treated cells with the Hippo signaling pathway inhibitor XMU-MP-1 for 4 days after cells were sub-cultured. XMU-MP-1 is a highly selective MST1/2 kinase inhibitor [24]. By directly targeting the kinase domain of MST1/2 core kinases in the Hippo signaling pathway, it inhibits the autophosphorylation of MST1/2 and subsequently inactivates LATS1/2. This process further blocks the phosphorylation of YAP/TAZ and promotes their nuclear translocation [25]. The results demonstrated that 5 μM XMU-MP-1 markedly down-regulated the expression level of CRX (Fig. 3C). 5 μM XMU-MP-1 did not significantly increase the cell death, but enlarged the cells (Supplementary Fig. 3). We further displayed that XMU-MP-1 significantly increased the nuclear retention ratio of YAP1 (Fig. 3D), decreased the amount of phosphorylated YAP1, and reduced the protein expression level of CRX (Fig. 3F, G), indicating that the Hippo/YAP1 pathway indeed inhibits CRX expression. Additionally, XMU-MP-1 treatment did not affect the nuclear retention ratio of CRX in ESC-RPE cells, indicating that the Hippo signaling pathway does not regulate the shuttling of CRX into the nucleus (Supplementary Fig. 4).

By employing transcription factor binding prediction software (TF-Target Finder, https://jingle.shinyapps.io/TF_Target_Finder/), we determined that TEAD has the potential to bind to the *CRX* promoter. Nevertheless, chromatin immunoprecipitation (ChIP) experiments demonstrated that neither TEAD nor YAP1 could directly bind to the *CRX* promoter region (Supplementary Fig. 5). This finding strongly indicates that YAP1 does not directly exert an inhibitory effect on CRX expression.

Previous studies reported that YAP1 can bind to β-catenin, promoting its nuclear retention, and can form a transcription complex with β-catenin and TCF to regulate gene expression [26, 27]. We found that in RPE cells with tight junctions, β-catenin is mainly distributed at the cell junctions (Fig. 3H). After sub-culturing, β-catenin is distributed in the cytoplasm and the nucleus (Fig. 3H). Co-immunoprecipitation (CoIP) experiments confirmed that YAP1 was able to bind to β-catenin, and when the Hippo signaling pathway is inhibited by XMU-MP-1, the entry of β-catenin into the nucleus increased significantly (Fig. 3I–K). MSAB is a specific β-catenin inhibitor that promotes β-catenin degradation [28], when RPE cells were treated with MSAB, the expression level of β-catenin decreased, and the expression level of CRX increased significantly (Fig. 3L–N). Moreover, 5 μM MSAB did not significantly increase the cell death (Supplementary Fig. 3). These results suggest that when the Hippo signaling pathway is inactivated, YAP1 enters the nucleus and promotes the nuclear retention of β-catenin, thereby inhibiting CRX expression.

To further validate the regulation of CRX gene expression by β-catenin, we utilized software to predict that TCF7 was capable of binding to the CRX promoter, and verified that by ChIP-quantitative real-time PCR (qRT-PCR) analysis (Fig. 3O–R). We further found that β-catenin bound to TCF7 (Supplementary Fig. 3). Upon knockdown of TCF7, the expression level of CRX increased significantly (Fig. 3T, U). These results demonstrated that β-catenin/TCF7 inhibits CRX expression.

These results confirm that β-catenin/TCF7 mediates the inhibitory effect of YAP1 on CRX expression. With the formation of tight junctions, the level of YAP1 in the nucleus gradually decreases, and the expression level of CRX increases.

### CRX suppresses EMT in RPE cells

**Fig. 4:**
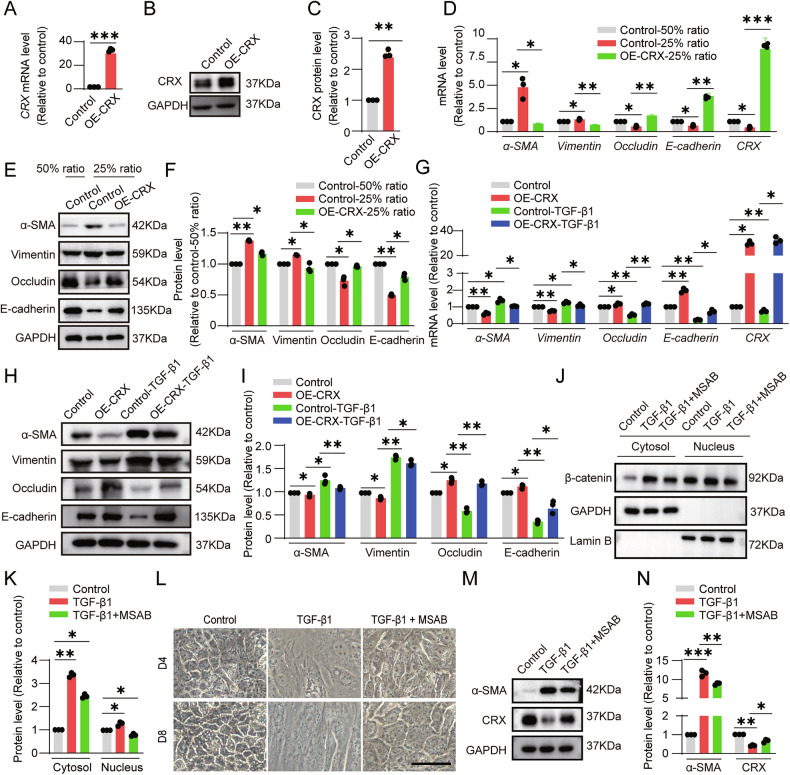
CRX inhibits EMT in ESC-RPE cells. CRX was overexpressed in ESC-RPE cells, and the expression level was detected by **A** qRT-PCR (*n* = 3), **B** Western blotting, and **C** quantitative analysis (*n* = 3). The expression levels of EMT-related genes in control- and OE-CRX-ESC-RPE cells after being sub-cultured for four days were determined by **D** qRT-PCR (*n* = 3), **E** Western blotting, and **F** quantitative analysis (*n* = 3). The sub-cultured control and OE-CRX-ESC-RPE cells were treated with 5 ng/mL TGF-β1 for 4 days. The expression levels of EMT-related genes were determined by **G** qRT-PCR (*n* = 3), **H** Western blotting, and **I** quantitative analysis (*n* = 3). **J**, **K** The protein level of β-catenin in the nucleus and cytoplasm of cells after being treated with 5 ng/mL TGF-β1 and 3 μM MSAB for four days was determined and quantified by Western blotting (*n* = 3). **L** Representative images of sub-cultured ESC-RPE cells treated with TGF-β1 and MSAB for 4 days and 8 days. **M**, **N** The expression levels of α-SMA and CRX in sub-cultured ESC-RPE cells treated with TGF-β1 and MSAB for 8 days were determined and quantified by Western blotting (*n* = 3). Scale bar = 50 μm. Data are presented as mean ± SD. Statistical significance was defined as follows: *p < 0.05, **p < 0.01, ***p < 0.001; (using unpaired two-sided *t*-tests in **B**, **C**, and one-way ANOVA and post hoc Bonferroni’s test in the others).

Previously, we discovered that ESC-RPE cells were more prone to undergo EMT when sub-cultured at a low density [13]. When we sub-cultured at a 25% ratio, we observed that ESC-RPE cells were not able to maintain their epithelial morphology after being cultured for 8 days compared with those with the sub-cultured at a 50% ratio, and discontinuous ZO-1 staining demonstrated the EMT phenotype (Supplementary Fig. 6A, B). The expression of α-SMA was significantly increased, whereas the expression of CRX was decreased (Supplementary Fig. 6C–F). To validate the function of CRX in EMT, we overexpressed CRX (OE-CRX) in ESC-RPE cells (Fig. 4A–C). On the 4th day of sub-culture, the overexpression of CRX inhibited the occurrence of EMT in cells, the expression of E-cadherin and occludin increased, and the expression of α-SMA and vimentin decreased (Fig. 4D–F).

During the formation of fibrotic scar, apart from the disruption of cell-cell junctions in RPE cells activating the YAP1 signaling pathway, another crucial inducer is TGF-β1 [29–31]. To further validate the function of CRX in resisting EMT, when sub-culturing cells, we immediately added TGF-β1 into the culture medium to induce EMT in cells to simulate the conditions for the fibrotic scar formation in vivo. The results showed that TGF-β1 treatment could reduce the expression levels of E-cadherin and occludin, while increasing the expression of α-SMA and vimentin (Fig. 4G–I). However, overexpression of CRX could counteract the effects of TGF-β1 (Fig. 4G–I). These results demonstrate that CRX can play a role in inhibiting EMT in RPE cells. Further experiments were conducted by overexpressing and knocking down CRX in iPSC-RPE cells to verify the role of CRX in inhibiting EMT in RPE cells (Supplementary Fig. 7).

Previously, we found TGF-β1 could promote β-catenin’s nuclear translocation in ARPE19 cells [9]. Similarly, in ESC-RPE cells, TGF-β1 facilitated the nuclear translocation of β-catenin (Fig. 4J, K); MSAB, in contrast, reduced this translocation (Fig. 4J, K). TGF-β1 induced EMT, increasing α-SMA and decreasing CRX expression (Fig. 4L–N). However, inhibiting β-catenin with MSAB increased CRX expression and inhibited EMT (Fig. 4L–N). These results demonstrated that TGF-β1 inhibits CRX expression, at least in part, through the β-catenin pathway.

CRX functions as a pivotal transcription factor in the differentiation process of photoreceptor cells [15]. Aiming to determine whether the overexpression of CRX in RPE cells instigates their differentiation into photoreceptor cells, the RNA-seq data was analyzed, photoreceptor-associated genes such as *RCVRN*, *RHO*, *NRL*, and *NR2E3* exhibited no substantial alterations. In contrast, RPE-associated genes, including *MITF*, *RPE65*, *RALBP1*, *TYRP1*, and *BEST1*, underwent up-regulation (Supplementary Fig. 8A). Subsequently, the gene expression levels were verified by qRT-PCR. The expression of RPE-related genes demonstrated an upsurge, whereas that of photoreceptor-related genes displayed a mixed pattern with overall low levels (Supplementary Fig. 8B). Based on our previously published chromatin immunoprecipitation sequencing (ChIP-seq) data (https://www.ncbi.nlm.nih.gov/geo, GSE174063) [14], it was evident that CRX binds to the promoter of *RPE65*, that was further validated by ChIP-qRT-PCR (Supplementary Fig. 8C, D). These data suggest overexpressing CRX in RPE cells does not induce their differentiation into photoreceptor cells. However, it holds the potential to enhance the expression of RPE associated genes, particularly that of RPE65, thereby enhancing the physiological functions of RPE cells.

### PPP2R2B mediates the inhibitory effect of CRX on TGF-β1-induced EMT

**Fig. 5:**
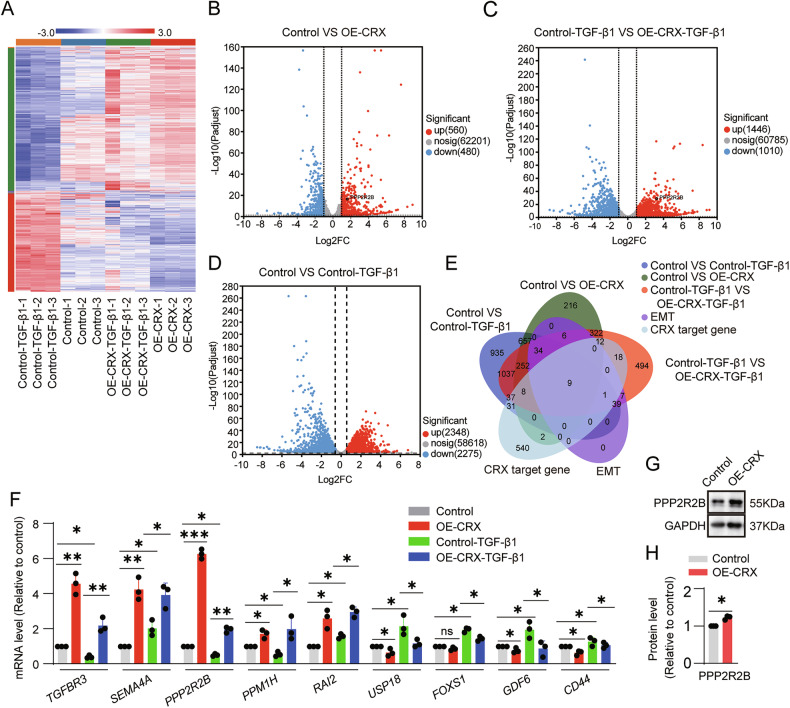
PPP2R2B is a downstream target of CRX. **A** Heatmap of RNA sequencing of control- and OE-CRX-ESC-RPE cells after being treated with TGF-β1. **B**–**D** Volcano plots of DEGs. **E** Nine target genes of CRX were screened out by venn analysis. The EMT gene set was extracted from the EMT pathway analysis, and the CRX target gene set was obtained from the TF_Target_Finder website (https://jingle.shinyapps.io/TF_Target_Finder/). **F** The expression levels of the 9 targeted genes were verified by qRT-PCR (*n* = 3). **G**, **H** The protein expression level of the target gene PPP2R2B in control- and OE-CRX-ESC-RPE cells was determined and quantified by Western blotting (*n* = 3). Data are presented as mean ± SD. Statistical significance was defined as follows: *p < 0.05, **p < 0.01, ***p < 0.001; ns, not significant (using one-way ANOVA and post hoc Bonferroni’s test in the (**F**) and unpaired two-sided *t*-tests in (**H**).

To elucidate the molecular mechanism by which CRX inhibits EMT in ESC-RPE cells, we conducted RNA-seq analysis on the normal control group (Control), the CRX overexpression group (OE-CRX), the TGF-β1-induced group (Control-TGF-β1), and the TGF-β1-induced OE-CRX group (OE-CRX-TGF-β1) (Fig. 5A). Volcano plots displayed the differentially expressed genes (DEGs) among different comparison groups (Fig. 5B–D). Through Venn analysis of the DEGs, we screened out 9 CRX target genes that related to EMT (Fig. 5E). The qRT-PCR detection determined that the expression level of *PPP2R2B* exhibited the most significant difference (Fig. 5F). *PPP2R2B* encodes a member of the protein phosphatase 2A (PP2A) B-subunit family [32]. The B-subunit serves as a structural component of the PP2A complex, enabling PP2A to interact with diverse substrates and execute various functions in different cellular processes [32]. We selected PPP2R2B as the target gene for our further study and found that overexpression of CRX in ESC-RPE cells promoted the expression of PPP2R2B (Fig. 5G, H).

**Fig. 6:**
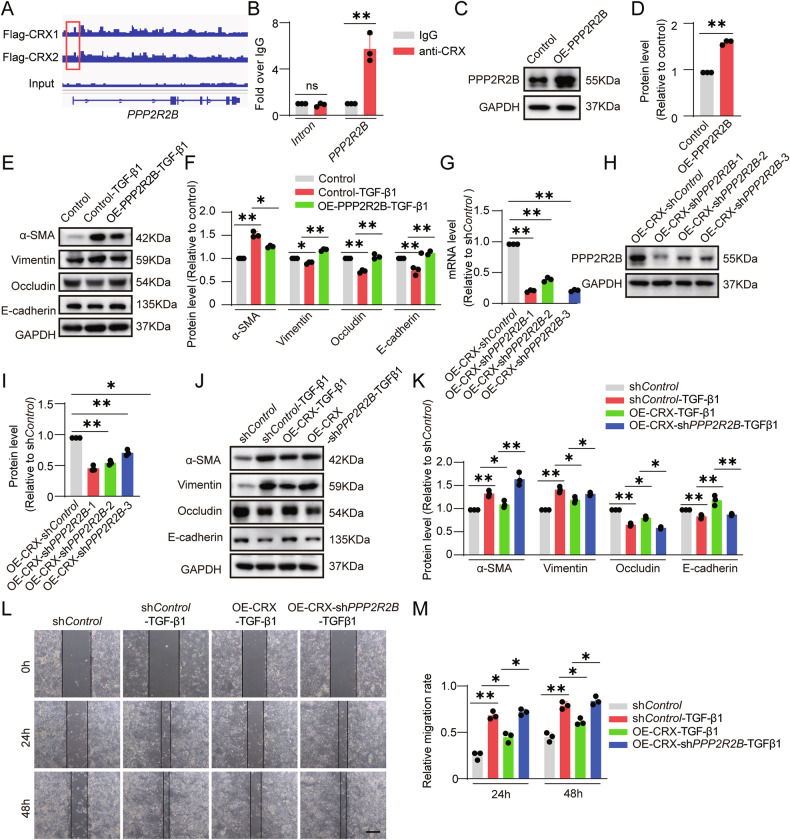
PPP2R2B mediates the effect of CRX in resisting EMT. **A** Enriched peaks of CRX binding to the promoter of *PPP2R2B*. **B** The fold enrichment of CRX immunoprecipitation relative to the IgG control, as determined by qRT-PCR (n = 3). **C**, **D** The overexpression of PPP2R2B in ESC-RPE cells was determined and quantified by Western blotting (*n* = 3). **E**, **F** The control- and OE-PPP2R2B-ESC-RPE cells were treated with TGF-β1, the expression levels of EMT-related proteins were determined and quantified by Western blotting (*n* = 3). PPP2R2B was knocked down in OE-CRX-ESC-RPE cells, and expression level of PPP2R2B was determined by **G** qRT- PCR (n = 3), **H** western blotting, and **I** quantitative analysis (*n* = 3). **J**, **K** Cells in different groups were treated with TGF-β1, the expression levels of EMT-related proteins were determined and quantified by Western blotting (*n* = 3). **L**, **M** Scratch assays were performed on cells in different groups treated with TGF-β1. The black lines represent the edges of the scratch. The cell migration rate was quantified as (distance between the black lines at the start of the experiment-distance between the black lines at the end of the experiment) / distance between the black lines at the start of the experiment (*n* = 3). Scale bar = 200 μm. Data are presented as mean ± SD. Statistical significance was defined as follows: *p < 0.05, **p < 0.01; ns, not significant (using unpaired two-sided *t*-tests in **B**, **D**, one-way ANOVA, and post hoc Bonferroni’s test in the others).

On the basis of on our previously published ChIP-seq data (https://www.ncbi.nlm.nih.gov/geo, GSE174063) [14], we next identified that CRX can bind to the promoter region of PPP2R2B, which was verified by ChIP-qRT-PCR (Fig. 6A, B). To explore the regulatory role of PPP2R2B in EMT of ESC-RPE cells, we first overexpressed PPP2R2B in ESC-RPE cells (Fig. 6C, D). Overexpression of PPP2R2B (OE-PPP2R2B) could partially counteract the alterations in EMT-related proteins induced by TGF-β1 (Fig. 6E, F). We then knocked down PPP2R2B in CRX-overexpressed ESC-RPE cells (OE-CRX-sh*PPP2R2B*) (Fig. 6G–I). We detected the knockdown efficiency of PPP2R2B by qRT-PCR and Western blotting, and selected OE-CRX-sh*PPP2R2B*-1 for subsequent experiments. Overexpression of CRX could inhibit the down-regulation of epithelial markers such as E-cadherin and occludin, as well as the increase in the expression of mesenchymal markers like α-SMA and vimentin induced by TGF-β1 (Fig. 6J, K). However, knockdown of PPP2R2B could counteract the inhibitory effect of CRX on EMT (Fig. 6J, K). Furthermore, through scratch assays, we found that TGF-β1 significantly enhanced the migratory capacity of ESC-RPE cells. Nevertheless, these effects were inhibited by CRX overexpression, and the inhibitory effect was counteracted by knockdown of PPP2R2B (Fig. 6L, M). The above results indicate that PPP2R2B exerts an inhibitory effect on EMT, and the anti-EMT effect of CRX is mediated, at least in part, by PPP2R2B.

### CRX reduced laser-induced CNV and subretinal fibrosis in mice

To evaluate the role of CRX in inhibiting EMT of RPE cells in vivo, we first established a laser-induced CNV mouse model. On the 3rd, 7th, and 21st days after laser induction, at the site of laser-induced lesion, the tissue is disorganized, and the structure of RPE cells was not able to be distinguished (Supplementary Fig. 9). The expression of CRX in RPE cells at the adjacent to the lesion site and distal to the lesion site was detected. The results showed that on the 3rd and 7th days, the number of RPE cells with positive CRX expression in the nucleus at the site adjacent to the lesion site and distal to the lesion site was significantly increased compared with the control group (Supplementary Fig. 9A, B). We further examined the expression of YAP1 and found that on the 3rd day and 7th days, a large number of RPE cells and choroidal cells at the site adjacent to the lesion site were positive for YAP1 nuclear expression. Thereafter, the number of RPE cells and choroidal cells with positive nuclear YAP1 expression gradually decreased (Supplementary Fig. 9A, B). Some RPE cells and choroidal cells in the distal area still had positive YAP1 nuclear expression at 7 and 21 days (Supplementary Fig. 9A, B). This result indicates that RPE layer at the lesion site was damaged by the laser, exhibited a disorganized tissue structure and loses its epithelial morphology, suggesting RPE cells may underwent EMT process. The RPE cells adjacent to the lesion site, similar to the slow-migrating cells in the scratch assay (Supplementary Fig. 2), expressed both CRX and YAP1 in the nucleus, indicating that CRX may play a role in inhibiting EMT. The RPE cells in the distal area were still affected by the microenvironment, with some RPE cells showing positive nuclear expression of both CRX and YAP1 (Supplementary Fig. 9A, B).

**Fig. 7:**
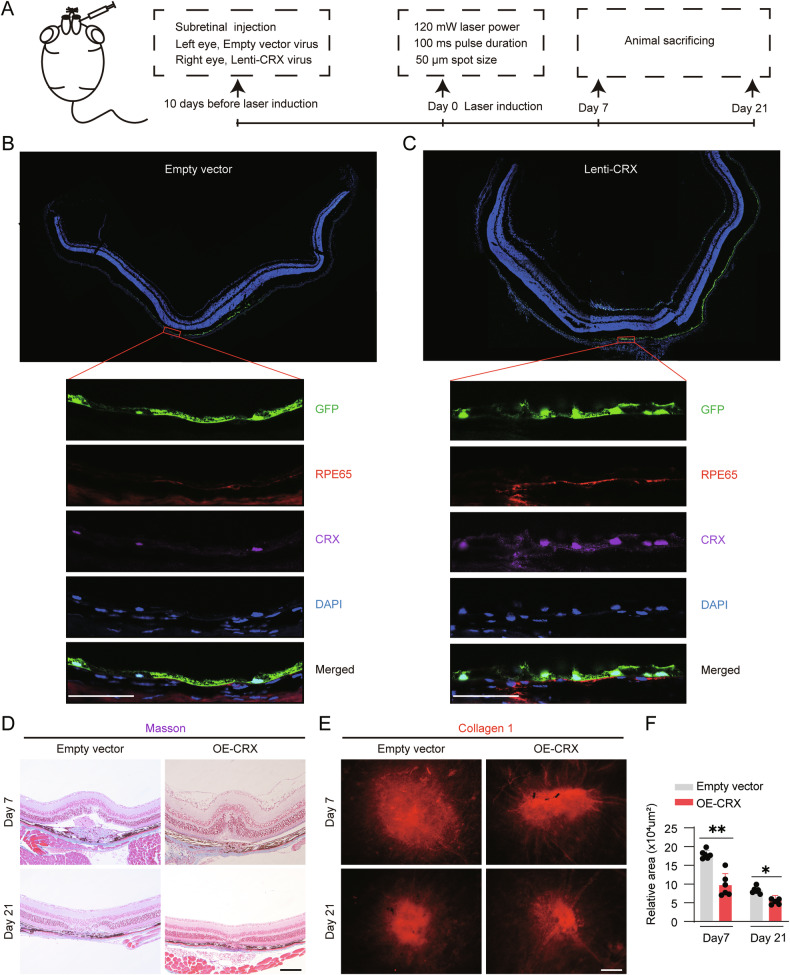
Overexpression of CRX in RPE cells reduces the area of laser-induced subretinal fibrosis in mice. **A** Schematic diagram of the laser-induced modeling and in vivo treatment in mice. Co-localization staining of GFP and CRX, RPE65 in the RPE cell layer 1 week after lentiviral infection in empty vector group (**B**) and lenti-CRX group (**C**). **D** Masson staining was performed to analyze the changes in the fibrotic area at different time points after laser induction. **E** Collagen 1 immunostaining demonstrated the fibrotic area in the choroidal flat-mount at different time points after laser induction. **F** The quantitative analysis of fibrotic area in the choroidal flat-mount (*n* = 3). Scale bar = 50 μm. Data are presented as mean ± SD. Statistical significance was defined as follows: *p < 0.05, **p < 0.01, not significant (using unpaired two-sided *t*-tests in (F).

Having previously confirmed the anti-EMT activity of CRX in vitro, we further investigated its therapeutic potential in vivo. We prepared lenti-CRX virus and administered it before laser photocoagulation via subretinal injection in the right eye, with the empty vector serving as the control in the left eye (Fig. 7A). Immunohistochemical analysis revealed predominant lentiviral transduction in RPE cells. Importantly, the CRX-overexpressing group demonstrated a significantly elevated proportion of CRX-positive RPE cells compared to empty vector controls, thereby confirming successful CRX overexpression in RPE cells (Fig. 7B, C). Retinal tissues were collected at postoperative days 7 and 21 following laser photocoagulation for subsequent analysis. Masson staining and immunostaining on the choroidal flatmounts were performed. Masson staining revealed that, compared to the empty vector group, significant disruptions were evident in the choroid and outer nuclear layer at the center of the laser burn group at 7 days after laser photocoagulation. Subsequently, 21 days after laser photocoagulation, retinal edema and newly-formed blood vessels extended into the subretinal space (Fig. 7D). In addition, compared with that in the empty vector group at days 7 and 21, the collagen area in the OE-CRX group was significantly reduced, indicating the reduced SF formation (Fig. 7E, F). We further did α-SMA immunostaining and found that the α-SMA+ area was markedly reduced (Supplementary Fig. 10) indicating the reduced EMT cells. These results suggest that the overexpression of CRX can inhibit EMT of RPE cells and reduce laser-induced SF in mouse retinas.

## Discussion

This study demonstrated that CRX functions as an endogenous signaling entity within RPE cells, effectively counteracting EMT. During the EMT process, the TGF-β1 and Hippo/YAP1 signaling cascades promote the nuclear translocation of β-catenin, which combines with TCF7 to bind to the promoter of CRX, thereby inhibiting CRX expression. Notably, overexpression of CRX leads to the up-regulation of PPP2R2B, which in turn suppresses EMT in RPE cells. The EMT-inhibitory efficacy of CRX has been experimentally validated in a laser-induced CNV mouse model.

During retinal development, CRX is considered a pivotal transcription factor implicated in photoreceptor cell fate determination [15]. Mutations in the human CRX gene are associated with several retinal disorders, including cone-rod dystrophy and Leber’s congenital amaurosis [33]. Previous studies posited that CRX is not expressed in RPE cells [18]. However, other research has detected the expression of CRX in human RPE cells [16, 17, 19]. Noriko Esumi not only confirmed the expression of CRX in human and bovine RPE cells but also discovered that CRX binds to the *BEST1* proximal promoter [17]. We further confirm that CRX is expressed in ESC-RPE and iPSC-RPE cells and can promote the expression of RPE65.

Previously, we found that transcription factors such as CRX, MITF, NR2E1, and cMYC could convert dedifferentiated RPE cells into iRPE cells with anti-EMT properties [14]. CRX can regulate the expression of multiple genes related to EMT[14]. In this study, we further discovered that CRX can promote the expression of PPP2R2B, a member of the PP2A B-subunit family. PP2A belongs to the Ser/Thr protein phosphatase family, which consists of a structural scaffold A-subunit, a highly conserved catalytic C-subunit, and diverse regulatory B-subunit [32]. The B-subunit serves as a structural component of the PP2A complex, enabling PP2A to interact with different substrates and execute various functions in diverse cellular processes [32]. Research has shown that PPP2R2B can inhibit EMT in bladder cancer cells by suppressing the WNT signaling pathway and reducing the expression of β-catenin [34]. PPP2R2B inhibits the proliferation and migration of pancreatic cancer cells by suppressing the mTOR pathway [35]. Using okadaic acid to inhibit PP2A can induce EMT in pancreatic tumor cells, and okadaic acid can strongly inhibit the expression of PPP2R2B [36]. PP2A inhibits the EMT process of podocytes by suppressing the JIP4/p38-MAPK pathway [37]. A significant amount of β-catenin is sequestered by E-cadherin at the plasma membrane, and knockdown of E-cadherin leads to the redistribution of β-catenin in the cytoplasm, followed by nuclear translocation and subsequent activation of the Wnt signaling [38]. We found that the overexpression of PPP2R2B promoted the expression of E-cadherin. It is highly likely that PPP2R2B inhibits the EMT process by increasing E-cadherin and reducing the nuclear translocation of β-catenin.

Crx functions as a transcription activator and cooperates with the bZIP transcription factor Nrl to activate the expression of the rhodopsin [18]. This finding indicates that a high level of rhodopsin expression demands the function of at least two photoreceptor transcription factors [18]. Additionally, both Crx and NeuroD1 are necessary to induce monkey iris-derived cells to acquire the photoreceptor phenotype [39]. These results imply that the interaction with other photoreceptor transcription factors is crucial for Crx to facilitate photoreceptor differentiation. This also accounts for the fact that in our study, merely overexpressing CRX in RPE cells did not lead to their transformation into photoreceptor cells. Instead, it even promoted the expression levels of RPE cell-specific genes, as evidenced by the significant increase in *RPE65*. Consequently, overexpressing CRX in RPE cells not only inhibits the occurrence of EMT in RPE cells but also holds the promise of enhancing the physiological functions of RPE cells.

During the development of CNV, new blood vessels in the choroid rupture Bruch’s membrane and grow beneath the RPE. These cells further extend into the subretinal space to form the SF, and the integrated RPE cells are damaged. The disruption of intercellular junctions inevitably inactivates the Hippo signaling pathway, leading to the activation and nuclear translocation of YAP1, thereby promoting the EMT of RPE cells [22, 23]. The expression level of YAP1 is significantly elevated in CNV tissues. Employing RNA interference technology to reduce the expression of YAP can decrease the expression of α-SMA in CNV tissues [40]. In the early stage of the laser-induced CNV model, we also observed the nuclear translocation of YAP1 in RPE cells.

Another crucial factor in inducing EMT in RPE cells is TGF-β1. Research has shown that the expression of TGF-β1 is significantly increased in the vitreous humor of wet AMD patients [29], and the amount of TGF-β1 is also remarkably elevated in the CNV model [30, 31]. The addition of TGF-β1 during RPE cell passage can rapidly promote EMT in cells. We found that YAP1 and TGF-β1 can synergistically inhibit the expression of CRX by promoting the nuclear translocation of β-catenin. β-catenin is a key molecule in the WNT signaling pathway, and its nuclear translocation can promote the EMT process [10]. Previously, we demonstrated that β-catenin can translocate into the nucleus under the stimulation of TGF-β1, promoting the EMT process in ARPE19 cells [9]. The decrease in CRX expression induced by TGF-β1 is also achieved through β-catenin. The transcriptional co-activator proteins YAP/TAZ are the hubs of the Hippo pathway. Although YAP/TEAD is not able to directly bind to the promoter region of CRX, YAP1 can also bind to β-catenin and promote its nuclear translocation. It has been reported that YAP1 interacts with and stabilizes the β-catenin protein in the nucleus [26], and YAP1 could directly interact with β-catenin in the nucleus and form a transcriptional YAP/β-catenin/TCF4 complex [27]. Our results are consistent with these reports. In addition, the Hippo signaling pathway can regulate the stability of β-catenin. We treated cells with XMU-MP-1, an inhibitor of the Hippo signaling pathway, and found that both the total expression level and nuclear translocation rate of β-catenin were increased. This result not only directly demonstrates that YAP1 promotes the nuclear translocation of β-catenin, but also confirms that the Hippo signaling pathway regulates the stability of β-catenin. Therefore, during the EMT process of RPE cells, signaling pathways such as TGF-β1, Wnt/β-catenin, and Hippo/YAP1 interact with each other and also have a synergistic regulatory effect on the expression of CRX.

The two principal pathogenic manifestations of wet AMD are MNV and SF [41–43] Anti-VEGF therapy has achieved encouraging outcomes in the treatment of MNV. However, there remains no effective approach for managing SF, which typically leads to permanent visual impairment [44, 45]. SF develops in the late stage of wet AMD. Multiple cell types, including RPE cells, macrophages, Müller cells, pericytes, and endothelial cells, transdifferentiate into myofibroblasts, promoting the formation of SF and collagen deposition [46–48]. Pathological analysis of surgically obtained MNV membranes revealed that the majority of cells were costained with α-SMA and cytokeratin. Since only RPE cells express cytokeratin, RPE cells represent important cell types within MNV membranes [6]. Thus, the EMT of RPE cells into myofibroblasts is one of the important sources of SF formation [46–48].

Numerous inhibitors targeting signaling pathways associated with RPE cell EMT have been developed to suppress the formation of SF. For example, knockdown of galectin-1 can inhibit the TGF-β1/Smad2/Snail signaling axis, thereby attenuating subretinal fibrosis in mice [42]. The expression of YAP1 is significantly elevated in CNV tissues. Employing RNA interference technology to reduce YAP1 expression can decrease the expression of α-SMA in CNV tissues [40]. RO4929097, a selective γ-secretase inhibitor, directly inhibits the Notch signaling pathway and indirectly suppresses the ERK1/2 signaling pathway in ARPE-19 cells and a laser-induced mouse model [49]. We have demonstrated that treatment with H535 (a β-catenin inhibitor) or Box5 (a Wnt5a inhibitor) effectively attenuates subretinal fibrosis and EMT in laser-induced CNV mice and ARPE19 cells [9]. This study reveals that CRX serves as an endogenous EMT-inhibitor within RPE cells that will provide novel research target for the treatment of wet AMD, especially in the prevention of the formation of SF.

Although we have elucidated that the Hippo/YAP1/β-catenin and TGF-β1/β-catenin pathways synergistically inhibit the expression of CRX, and the nuclear translocation of CRX is not regulated by the Hippo signaling pathway, the signaling pathway that regulates the nuclear shuttling of CRX remains unclear. In addition, the TGF-β signaling pathway consists of both canonical and non-canonical signaling pathways [50]. Whether the canonical SMAD2/3 signaling pathway also regulates CRX expression requires further verification. Furthermore, age can affect the expression of multiple signaling molecules involved in this study. For instance, the expression of YAP1 changes with increasing age, and overexpression of YAP1 can inhibit cellular senescence [51]. The Wnt/β-catenin signaling pathway is significantly induced in aged mice and plays a crucial role in age-related mitochondrial dysfunction and organ fibrosis [52]. Additionally, the expression of PPP2R2B is downregulated in tumor cells with high proliferative activity, and overexpression of PPP2R2B can inhibit cell proliferation and migration [53]. In the present study, 8-week-old young mice were used as experimental animals. Although young mice can avoid the interference of other age-related factors, allowing us to focus on the molecular mechanism by which CRX inhibits EMT, study have shown that aged mice are more prone to CNV model establishment and better simulate the pathogenesis of AMD in patients[47]. Therefore, it is necessary to establish a CNV model in aged mice to confirm the inhibitory effect of CRX on EMT.

**Fig. 8:**
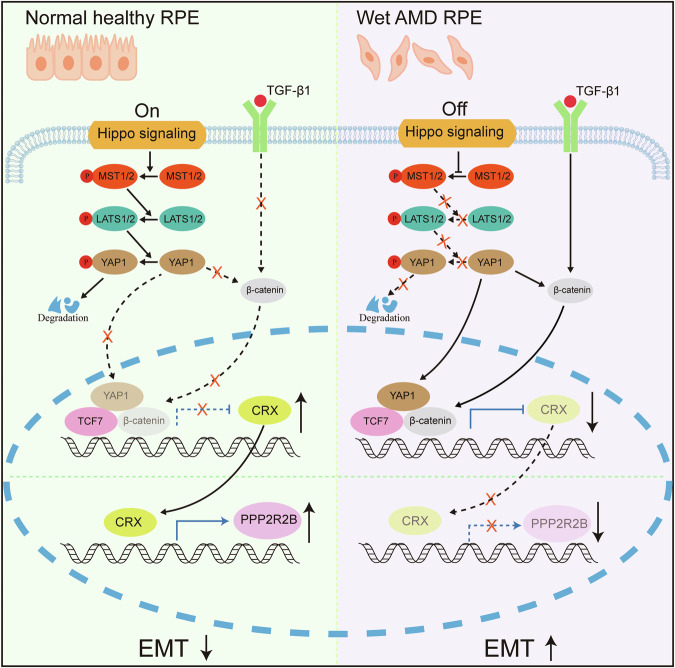
The Hippo/YAP1 and TGF-β signaling pathways synergistically inhibit the expression of CRX. In normal and healthy RPE cells, the Hippo signaling pathway is activated. Under this condition, YAP1 is phosphorylated and subsequently degraded, while the TGF-β signaling pathway remains inactivated. The translocation of β-catenin into the nucleus is blocked, leading to the activation of CRX expression. This activation of CRX then promotes the expression of PPP2R2B and, in turn, inhibits the EMT. Conversely, in the RPE cells of wet AMD, the Hippo signaling pathway is inactivated, and the phosphorylation of YAP1 is blocked. This inactivation of the Hippo pathway, in synergy with the activated TGF-β signaling pathway, promotes the translocation of β-catenin into the nucleus. β-catenin binds to TCF7, which results in the inhibition of CRX expression. Subsequently, the repression of PPP2R2B expression promotes EMT.

In summary, this study utilized the published single-cell sequencing data to confirm the expression of CRX in human RPE cells and verified it in vitro-cultured ESC-RPE and iPSC-RPE. Sub-culturing was used to disrupt intercellular connections and thus inhibit the Hippo signaling pathway, combined with TGF-β1 treatment to simulate the microenvironment for the SF formation. It was found that Hippo/YAP1 and TGF-β1 synergistically promoted the nuclear translocation of β-Catenin, and the latter combined with TCF7 to bind to *CRX* promoter to inhibit the expression of CRX (Fig. 8). Overexpression of CRX could suppress the EMT of RPE cells by up-regulating the expression of PPP2R2B. In the laser-induced mouse CNV model, the nuclear translocation of CRX also occurred in RPE cells, and overexpression of CRX played an inhibitory role in the formation of subretinal fibrosis. This study identified CRX in RPE cells as an endogenous signaling molecule that inhibits EMT, providing a new treatment strategy and research target for the treatment of wet AMD and the prevention of SF formation. This study also offers a new treatment method for other ophthalmic diseases with EMT of RPE as the critical pathogenesis, such as proliferative vitreoretinopathy, diabetic retinopathy, and inherited retinal degeneration[54–56].

## Materials and methods

### Cell cultures

The SHhES2 human embryonic stem cell (ESC) line was a gift from Professor Y. Jin (Institute of Health Sciences, Chinese Academy of Sciences, Shanghai, China). Human induced pluripotent stem cells (iPSCs) were established by our lab [14]. Differentiation of ESCs and iPSCs into ESC-RPE cells and iPSC-RPE cells was conducted according to our previously described protocol [14]. Briefly, once growing to confluency, ESCs and iPSCs were cultured in differentiation medium containing DMEM/F12, 15% knockout serum replacement (KSR, Gibco), 1% nonessential amino acids, 2 mM glutamine, 50 U/ml penicillin, 50 mg/ml streptomycin (all from Invitrogen, Carlsbad, CA), and 10 mM nicotinamide (Sigma) in 6-well culture dishes (Costar, Corning Inc., Corning, NY) pretreated with Poly (2-hydroxyethyl methacrylate) (Sigma). 20 ng/ml Activin A (PeproTech Inc., Rocky Hill, NJ) was supplemented during the 3rd and 4th weeks. After 8 weeks in suspension, pigmented areas were isolated by a surgical blade no. 15 and 30–50 clusters were cultured in dishes precoated with Matrigel and cultured 6 weeks in differentiation medium. ESC-RPE and iPSC-RPE were passaged with 1:2 ratio and cultured in medium DMEM/F-12, 10% KSR, supplemented with 2 mM L-glutamine, 1% MEM nonessential amino acids (MEM NEAA, Gibco),1% penicillin-streptomycin, 0.1 mM 2-mercaptoethanol (Gibco), and 10 mM nicotinamide in dishes precoated with 1% Matrigel at 37 °C and 5% CO_2_. The RPE cells at passages 3–5 were used in this study.

### Analysis of the single-nuclei RNA-seq data of retinal tissues

The single-nuclei RNA-seq data of 106 human retina and RPE/choroid with subtype resolution from over 100,000 cells (including the information of cell types) were obtained from GEO (https://www.ncbi.nlm.nih.gov/geo/) database (GSE135133). The merged expression matrix was prepared for clustering using the Seurat 4.4.0, following the common pipeline. Uniform Manifold Approximation and Projection (UMAP) dimensionality reduction was used to project cells in two dimensions. The function FeaturePlot and VlnPlot in Seurat were used to visualization the gene expressions.

### Analysis of microarray data

The microarray data of normal human RPE/choroid and neural retina (with the age of all the individuals being over 75) were obtained from the GEO (https://www.ncbi.nlm.nih.gov/geo/) database (GSE29801). The relative level of *CRX* in each group was calculated.

### Immunostaining

For immunostaining, fixed cells and cryosections from eyes were permeabilized with 0.25% Triton X-100 (Sigma) for 2 min, washed with PBS, and then blocked with 2% bovine serum albumin (BSA, Sigma) in PBS. The samples were incubated with the primary antibodies against α-SMA (Abcam, Cambridge, UK), ZO-1 (Proteintech, Chicago, USA), CRX (Proteintech), YAP1 (Proteintech), β-catenin (Proteintech), and RPE65 (Novus Biologicals, Centennial, CO, USA) overnight at 4 °C. They were then washed three times with PBS, followed by incubation with the fluorescent secondary antibodies overnight. 4,6 diamidino-2-phenylindole dihydrochloride (DAPI, Sigma) was used to indicate the nucleus. The samples were then examined by fluorescence microscope (Olympus IX73, Tokyo, Japan) or LSM 900 confocal microscope system (Zeiss, Germany). The nuclear retention rate of CRX and YAP1 was determined as (number of CRX + RPE cells or YAP1+ cells) / (number of DAPI + RPE cells plus number of DAPI+ choroidal cells) × 100%. Antibodies were listed in Table S1.

### Quantitative real-time PCR (qRT-PCR)

Total RNA was extracted, and reverse transcription was performed using Primescript™ RT Master Mix kit (Takara, Shiga, Japan). qRT-PCR was performed in a Chromo4 instrument cycler (Bio-Rad, Hercules, USA) using Superreal Premix plus kit (Tiangen Biotech, Beijing, China). PCR amplification was carried out with the following cycling parameters: denaturation at 95 °C for 5 min, followed by 40 cycles of 95 °C for 10 s, 60 °C for 30 s. Primer sequences (Synthesized by Sangon Biotech, China) were listed in Table S2.

### Western blotting

The cells were lysed by RIPA buffer containing protease and phosphatase inhibitor (Sigma). The protein extracts (20 μg per sample) were separated by 10% SDS-PAGE gels, and transferred onto polyvinylidene difluoride membranes (Millipore, Bedford, MA, USA). After blocked with 3% BSA in PBS for 1 h, membranes were incubated with primary antibodies against α-SMA, E-cadherin (Cell Signaling Technology, Beverly, MA, USA), Vimentin (Proteintech), YAP1, phospho-YAP1(Ser397) (Proteintech), β-catenin, CRX, TCF7 (Cell Signaling Technology), PPP2R2B (Abcam), and β-actin or GAPDH (Proteintech) for 12 h at 4 °C, followed by incubation with corresponding secondary antibodies for 1 h at room temperature. The blots were visualized with a chemiluminescence imaging system (Tanon 5200, Shanghai, China) and quantified with ImageJ software (Version 1.48v). Antibodies were listed in Table S1.

### Co-immunoprecipitation (Co-IP)

Protein A + G magnetic beads (20 μL) were incubated with 5 μg antibodies (against TCF7, β-Catenin) or normal 5 μg IgG for 1 h. 5 × 10^6^ cells were lysed by 200 μL IP lysis buffer (Beyotime, Shanghai, China) and incubated with prepared conjugated Protein A + G magnetic beads at 4 °C overnight. The beads were washed with TBS for three times, and the binding proteins were eluted with protein loading buffer at 95 °C for 5 min. After centrifugation, the samples were collected and used for Western blotting.

### Chromatin immunoprecipitation-quantitative PCR (ChIP-qPCR) assay

The ChIP assay was performed using a SimpleChIP Plus Enzymatic Chromatin IP kit (Cell Signaling Technology). Briefly, ten million cells were treated with 1% formaldehyde for 10 min, and unreacted formaldehyde was quenched with glycine for 5 min. The cells were collected by scraping and were lysed with lysate buffer; the nuclear pellet was digested by micrococcal nuclease. Immunoprecipitation was performed on the lysate with 2 μg antibody against YAP1, β-catenin, TCF7, CRX, or control rabbit IgG at 4 °C overnight. Then, the immune complexes were incubated with Protein G magnetic beads (Cell Signaling Technology) for 2 h. Chromatin was eluted from antibody/protein G magnetic beads and used for qRT-PCR. The ChIP-qPCR primer sequences for the promoters of CRX, PPP2R2B, and RPE65 are summarized in Table S3.

### Scratch assay

Cells were plated into 24-well culture plate and grow to confluency. Then, scratches on ESC-RPE cells monolayers were made with a sterilized 200-μL pipette tip and then gently washed twice with sterile PBS to remove floating debris. Images were recorded after 0, 24, and 48 h, and the wound recovery was analyzed using ImageJ software.

### Overexpression of CRX and PPP2R2B

For generating lentivirus, human cDNAs of CRX and PPP2R2B were obtained by PCR amplification from ESC-RPE cells and cloned into lentiviral pLVX-mCMV-ZsGreen1-Puro vector (Takara). The packaging plasmids were psPAX2 and pMD2.G. HEK293FT cells were seeded at a density between 5.0 and 7.0 × 10^4^ cells/cm^2^ and transfected by Lipofectamine 2000 (Invitrogen) with each vector. Individual supernatants containing virus were harvested at 48 h post-transfection and filtered with a 0.45 μm PVDF membrane (Millipore, Boston, USA). ESC-RPE cells and iPSC-RPE cells were plated in 6-cm culture dishes, respectively. The next day, cells were infected with viruses. The positively transfected cells were sorted by FACS based on ZsGreen expression. The expression levels were determined by qRT-PCR and Western blotting.

### Generation of lentiviral vector to knockdown target genes

HEK293T cells were seeded in 15 cm dishes at a density of 10^7^ cells per dish 24 h before transfection. Lentiviral pLVX-shRNA2-ZsGreen1 (Takara) vector was used to prepare lentiviruses; the packaging plasmids are psPAX2 and pMD2.G. HEK293FT cells were transfected with vectors. Individual supernatants containing virus were harvested at 48 h and used to infect cells. Reduced expression of target genes at the transcript level was determined by qRT-PCR. The targeting sequences of shRNAs for *CRX*, *TCF7*, and *PPP2R2B* were included in Table S4. The most effective shRNA sequence was selected.

### RNA sequencing

Following the Illumina mRNA-seq protocol, pooled RNA libraries of the cells were established, with 50 ng of RNA from RPE cells from different groups. Sequencing was performed by the MAJORBIO company (Shanghai, China). Filtering and quality control of the raw reads from RNA-seq was carried-out using FastQC. The clean reads were mapped to reference sequences using SOAP2 aligner. Gene expression levels were calculated using the TPM method. Log_2_ fold change (FC) of TPM was used to identify differentially expressed genes (DEGs) between these two groups. Only those genes indicating log |FC| > 1 and adjusted *p* < 0.05 were regarded as DEGs.

### Calcein/PI staining

Cells were cultured in 96-well culture plate. 1–5 μM XMU-MP-1 or MSAB (MedChemExpress, Shanghai, China) was used to treat ESC-RPE cells. For Calcein/PI staining, cells were cultured in 100 μL of calcein AM/PI working solution (Beyotime) at 37 °C for 1 h. The samples were then examined by a fluorescence microscope (Olympus IX73)

### Animals

In this study, 8-week-old C57BL/6J male mice (from the Laboratory Animal Center of Tongji University) were utilized. They were housed in groups and were allowed a period to acclimatize to the laboratory environment with a 12/12 h light/dark cycle and provided with food and water.

### Subretinal injection of virus and laser-induced CNV model

A total of 40 C57BL/6J male mice were divided into laser-empty vector groups and laser-overexpression of CRX (OE-CRX) groups. Mice were firstly anaesthetized by 1.25% tribromoethanol, their pupils were dilated with amethocaine (0.5%) and tropicamide (0.5%). A channel was created by inserting a 30-gauge needle behind the limbus into the vitreous chamber. A 33-gauge needle was inserted into the subretinal space of the central retina, and 2 μL lentivirus carrying the *CRX* gene was injected. The control eyes received an injection of lentivirus carrying an empty vector. Seven days after subretinal injection of virus, the laser-induced subretinal fibrosis model was established as previously described [9, 57–59]. In brief, four to six laser spots (532 nm, 120 mW, 100 ms; Novus Spectra, Japan) were selected at each fundus around the optic disc and avoid tiny blood vessels. The disruption of Bruch’s membrane was confirmed by visually subretinal bubble formation immediately after laser application. After laser burns, the mice were randomly sacrificed on day 7 and day 21 for further quantification of CNV and subretinal fibrosis.

### Masson’s trichrome staining

The eyes were obtained at days 7 and 21 after laser induction. The samples were fixed with 4% PFA and embedded in paraffin to prepare the 3 µm-thick sections. Masson’s trichrome staining was performed with a trichrome staining kit (Wuhan Servicebio Technology Co., Ltd., Wuhan, China) according to the manufacturer’s protocol. Collagen fibers were stained blue, and images were obtained by microscope (Olympus IX73).

### RPE/choroid flatmount staining

The areas of CNV and collagen fibers were determined on RPE/choroidal flatmounts on day 7 and day 14. Mouse eyecups were fixed in 4% Paraformaldehyde (PFA), and anterior segments were removed before cutting six to eight radial incisions to be flattened. The RPE-choroid complexes were washed, blocked with 5% BSA, and permeabilized with 0.3% Triton X-100, and then incubated with collagen type I antibody (Abcam) (for evaluating subretinal fibrosis) at 4 °C overnight. They were washed three times with PBS, followed by incubation with the fluorescent secondary antibodies overnight. Samples were finally observed under fluorescence microscope (Olympus IX73).

### Statistical analysis of data

All values are expressed as the mean ± SD. Data were analyzed via GraphPad Prism 9 software (GraphPad Software, San Diego, CA, USA). Differences between two groups were assessed with the two-tailed Student’s unpaired *t*-test. The one-way ANOVA and post hoc Bonferroni’s test were used to assess differences between more than two groups. No statistical methods were used to predetermine the sample size. Mice were randomly allocated to experimental groups, and no blinding method was used for subretinal injection. Eyes with damaged lens or damaged retina due to subretinal injection were excluded from experiments. The variance was similar between the groups that were being statistically compared. All experiments were repeated twice. Statistical significance thresholds were set at ns. insignificant, *p < 0.05, **p < 0.01, ***p < 0.001.

## Supplementary information

Supplementary Figure Legends

Supplementary Figure 1

Supplementary Figure 2

Supplementary Figure 3

Supplementary Figure 4

Supplementary Figure 5

Supplementary figure 6

Supplementary Figure 7

Supplementary Figure 8

Supplementary figure 9

Supplementary Figure 10

Supplementary Tables

Western blotting original figures

## Data Availability

All study data are included in the main text and/or SI Appendix. The raw RNA-seq data have been deposited in the NCBI Sequence Read Archive (SRA) database under accession number: PRJNA1169190.
