# Supplementary material for: CRX is an intrinsic suppressor of epithelial‒mesenchymal transition in retinal pigment epithelial cells: a promising therapeutic avenue for subretinal fibrosis
Source: Cell Death Dis. 2025 Dec 31;17(1):156. doi: 10.1038/s41419-025-08352-y (PMC12859066; doi:10.1038/s41419-025-08352-y)
Supplement: Supplementary file 1 — Supplementary Figure Legends [file 41419_2025_8352_MOESM1_ESM.docx]

**Supplementary Fig. 1.** RPE cells express CRX. **A** CRX expressing in ESC-RPE cells and iPSC-RPE cells was demonstrated by Western blotting. **B** The expression of CRX in human neural retina and RPE cells + choroid was confirmed by the gene microarray data from healthy people (n = 7).

**Supplementary Fig. 2** ESC-RPE cells initiate the EMT following scratching. **A** Subsequent to scratching, ESC-RPE cells commenced migration and entered the EMT process. The white arrows denote the direction of migration, while the black arrows respectively indicate the slow-migrating cells and the disseminated cells. **B** Immunostaining for CRX and α-SMA demonstrated that both the slow-migrating cells and the disseminated cells express α-SMA. However, seven days after scratching, the disseminated cells no longer expressed CRX. **C** Seven days post-scratching, ZO-1 staining revealed that the disseminated cells completely lost their polygonal morphology, which is a clear indication that they have undergone EMT. **D** Immunostaining for CRX and YAP1 demonstrated that both the slow-migrating cells and the disseminated cells expressed YAP1. Scale bar = 50 μm.

**Supplementary Fig. 3** XMU-MP-1 and MSAB are not toxicity to ESC-RPE cells. **A** Calcein/PI staining of ESC-RPE cells treated with 1-5 μM XMU-MP-1 or MSAB. The white dotted circle demonstrated the cell size. **B** The ratio of PI+ cells was used to quantify the death of ESC-RPE cells (n = 8). Data are presented as mean ± SD. Statistical significance was defined as follows: ns, not significant (using one-way ANOVA and post hoc Bonferroni’s test).

**Supplementary Fig. 4** XMU-MP-1 treatment does not after the nuclear shuttling of CRX. **A** After treatment with 5 μM XMU-MP-1 for one day, the nuclear retention of CRX was detected by immunostaining. **B** The quantitative analysis of CRX nuclear retention ratio (n = 8). Data are presented as mean ± SD. Statistical significance was defined as follows: ns, not significant (using unpaired two-sided t-tests).

**Supplementary Fig. 5** The verification of YAP1 and TEAD2 binding to the promoter of CRX. Fold enrichment of YAP1 and TEAD2 immunoprecipitation compared with IgG control, as determined by qRT-PCR (n = 3). Data are presented as mean ± SD. Statistical significance was defined as follows: ns, not significant (using unpaired two-sided t-tests).

**Supplementary Fig. 6** ESC-RPE cells undergo EMT when sub-cultured at a low density. **A** Representative image of ESC-RPE cells sub-cultured at a 50% ratio and a 25% ratio after 4 days and 8 days of culture. **B** The expression of ZO-1 at different time points during the cell sub-culturing process. **C-D** The expression levels of (C) α-SMA and (D) CRX were determined by qRT-PCR (n = 3). **E-F** The expression levels of α-SMA and CRX were determined and quantified by Western blotting (n = 3). Data are presented as mean ± SD. Statistical significance was defined as follows: *p < 0.05, **p < 0.01, ***p < 0.001 (using unpaired two-sided t-tests and one-way ANOVA and post hoc Bonferroni’s test).

**Supplementary Fig. 7** CRX exerts an anti-EMT effect in iPSC-RPE cells. **A-C** CRX was overexpressed in iPSC-RPE cells, (**A**) QRT-PCR and (**B**) Western blotting were used to detect its mRNA and protein levels, along with (**C**) quantitative calculation (n = 3). **D-F** CRX was knocked down in iPSC-RPE cells, (**D**) qRT-PCR and (**E**) Western blotting were used to detect its mRNA and protein levels, along with (**F**) quantitative calculation (n = 3). **G-I** Four days after sub-culturing cells with 50% or 25% ratio, the EMT-related genes and proteins were determined by (**G**) qRT-PCR, (**H**) Western blotting, and (**I**) quantitative analysis (n = 3). **J-K** The sub-cultured control- and OE-CRX-iPSC-RPE cells were treated with 5 ng/mL TGF-β1 for four days, the EMT-related proteins were determined by Western blotting and quantitative analysis (n = 3). Data are presented as mean ± SD. Statistical significance was defined as follows: *p < 0.05, **p < 0.01, ***p < 0.001 (using unpaired two-sided t-tests and one-way ANOVA and post hoc Bonferroni’s test).

**Supplementary Fig. 8** Overexpression of CRX in ESC-RPE cells does not promote their differentiation into photoreceptor cells. **A** RNA-seq data were used to analyze the TPM values of RPE-related markers (*RPE65*, *BEST1*, *TYRP1*, *RALBP1*, *MITF*, and *OTX2*) and photoreceptor-related markers (*RHO*, *RCVRN*, *NR2E3*, *NRL*, *RS1*, *RORB*, *ASCL*, *THRB*, and *RXRG*) in the control group and the OE-CRX group (n = 3). **B** RPE-related markers and photoreceptor-related markers were determined by qRT-PCR (n = 3). **C-D** Enriched peaks of CRX binding to the promoter of *RPE65* and the fold enrichment of CRX immunoprecipitation compared with the IgG control, as determined by qRT-PCR (n = 3). Data are presented as mean ± SD. Statistical significance was defined as follows: *p < 0.05, **p < 0.01, ***p < 0.001(using unpaired two-sided t-tests).

**Supplementary Fig. 9** Expressions of CRX and YAP1 in RPE cells and choroidal cells of laser-induced CNV mice. **A** The nuclear retention of CRX and YAP1 at different time points in RPE cells and choroidal cells of laser-induced CNV mice. The arrows point the laser lesion site, proximal to lesion site, and distal to lesion site. RPE cells and choroidal cells layer is between the yellow dotted lines and pointed by arrow. **B** The quantitative analysis of the nuclear retention of CRX and YAP1 (n = 7). Scale bar = 50 μm. Data are presented as mean ± SD. Statistical significance was defined as follows: **p < 0.01, ***p < 0.001; (using one-way ANOVA with post hoc Bonferroni correction).

**Supplementary Fig. 10** Overexpression of CRX in RPE cells reduces the area of laser-induced subretinal fibrosis in mice. **A** α-SMA immunostaining demonstrated the fibrotic area in the choroidal flat-mount at day 7 after laser induction. **B** The quantitative analysis of fibrotic area in the choroidal flat-mount (n = 6). Scale bar = 50 μm. Data are presented as mean ± SD. Statistical significance was defined as follows: **p < 0.01(using unpaired two-sided t-tests).
