# Supplementary figures and images for: CRX is an intrinsic suppressor of epithelial‒mesenchymal transition in retinal pigment epithelial cells: a promising therapeutic avenue for subretinal fibrosis
Source: Cell Death Dis. 2025 Dec 31;17(1):156. doi: 10.1038/s41419-025-08352-y (PMC12859066; doi:10.1038/s41419-025-08352-y)

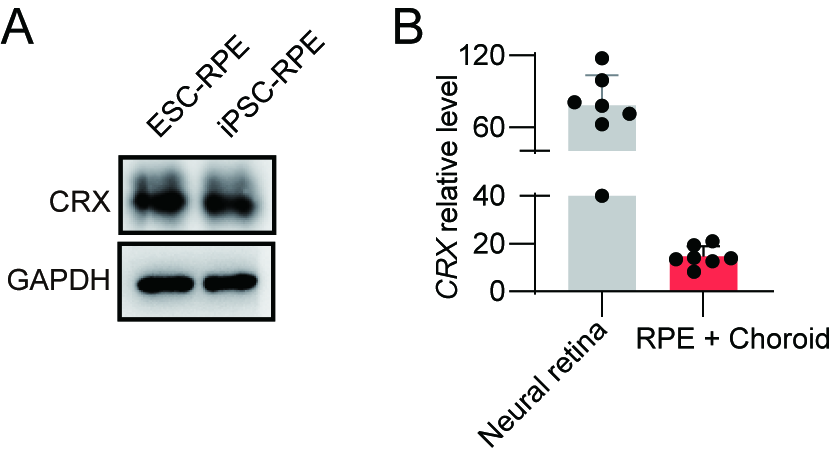

Supplement: Supplementary file 2 — Supplementary Figure 1 [file 41419_2025_8352_MOESM2_ESM.tif]

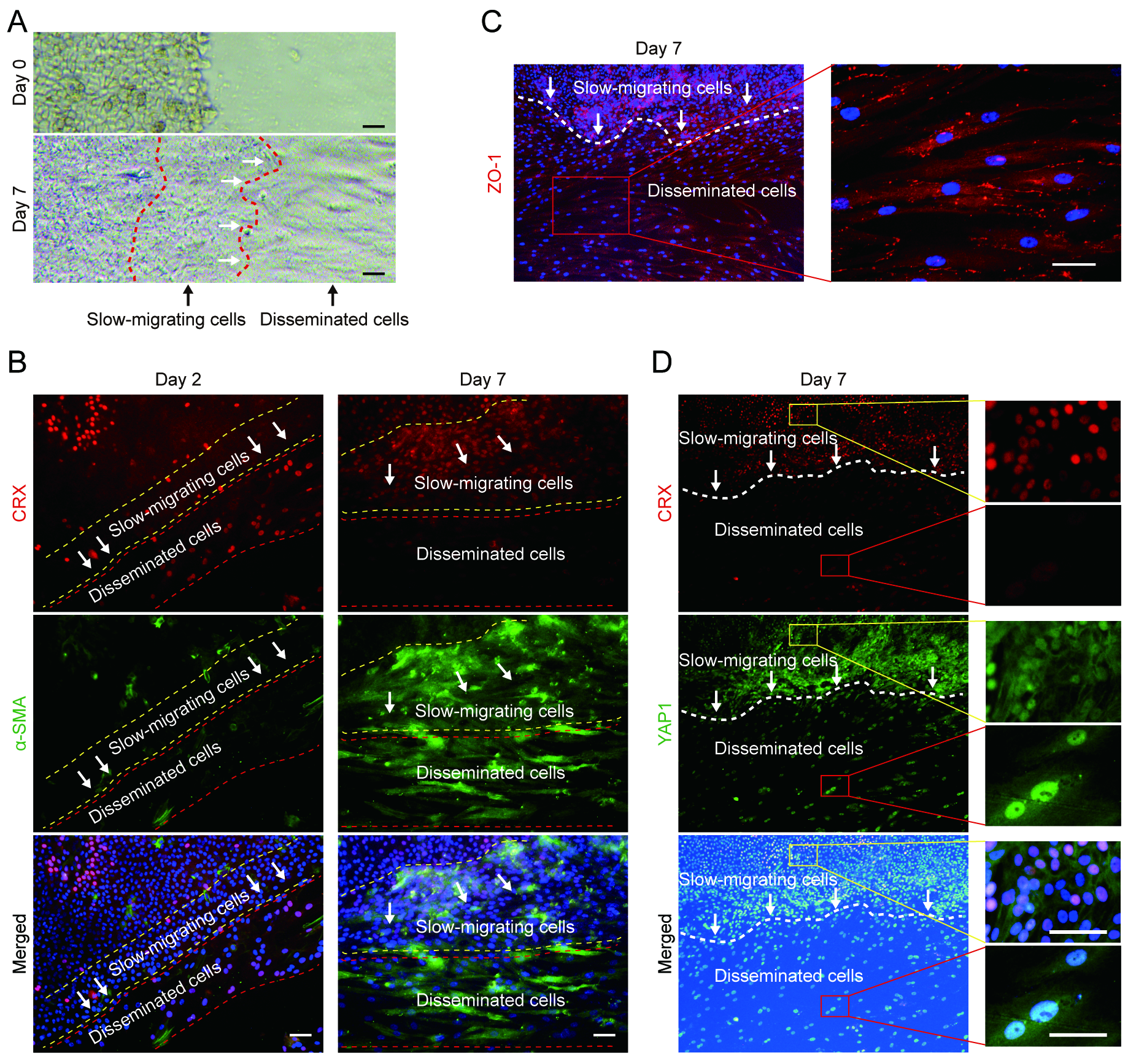

Supplement: Supplementary file 3 — Supplementary Figure 2 [file 41419_2025_8352_MOESM3_ESM.tif]

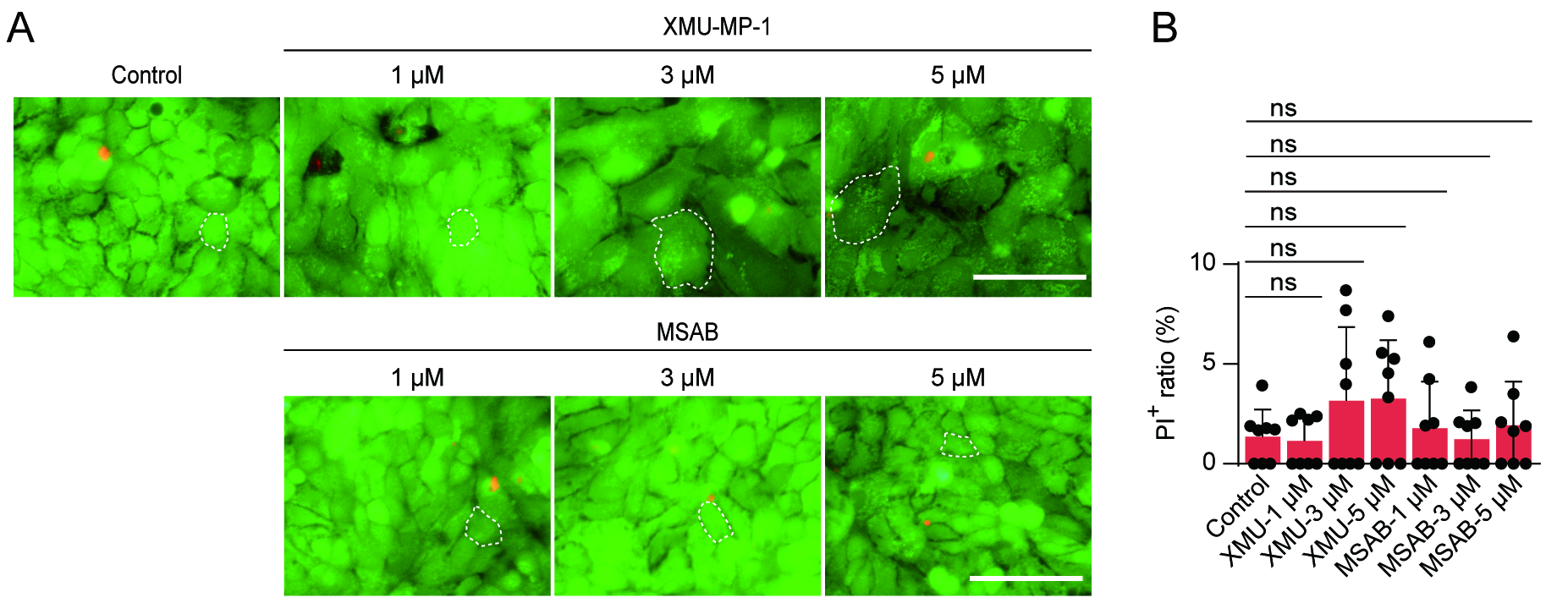

Supplement: Supplementary file 4 — Supplementary Figure 3 [file 41419_2025_8352_MOESM4_ESM.tif]

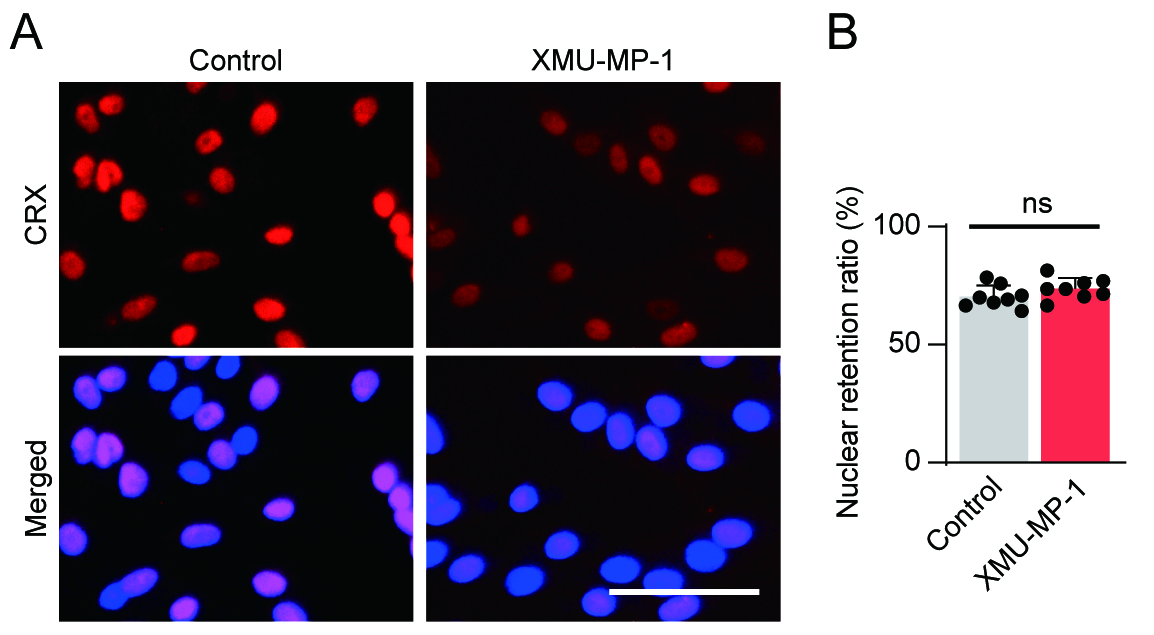

Supplement: Supplementary file 5 — Supplementary Figure 4 [file 41419_2025_8352_MOESM5_ESM.tif]

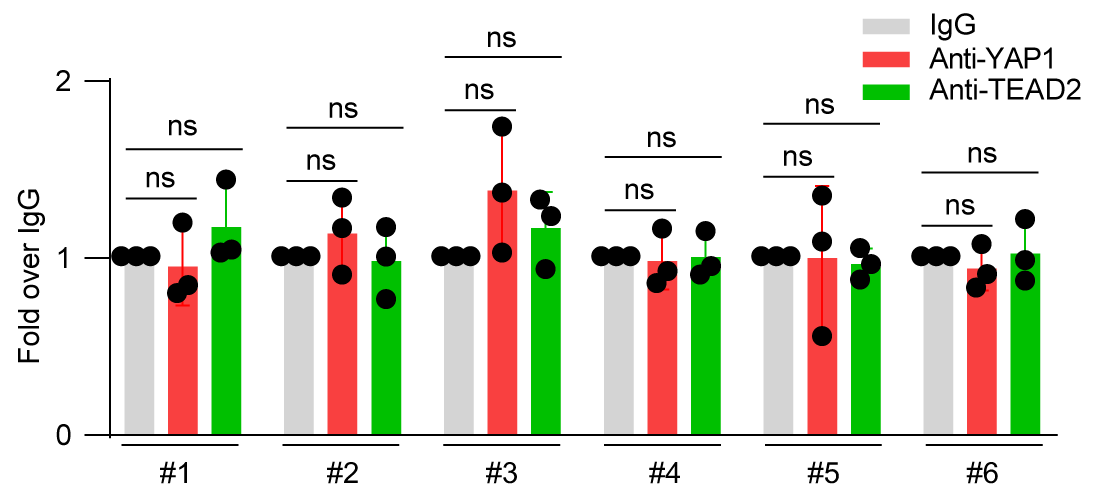

Supplement: Supplementary file 6 — Supplementary Figure 5 [file 41419_2025_8352_MOESM6_ESM.tif]

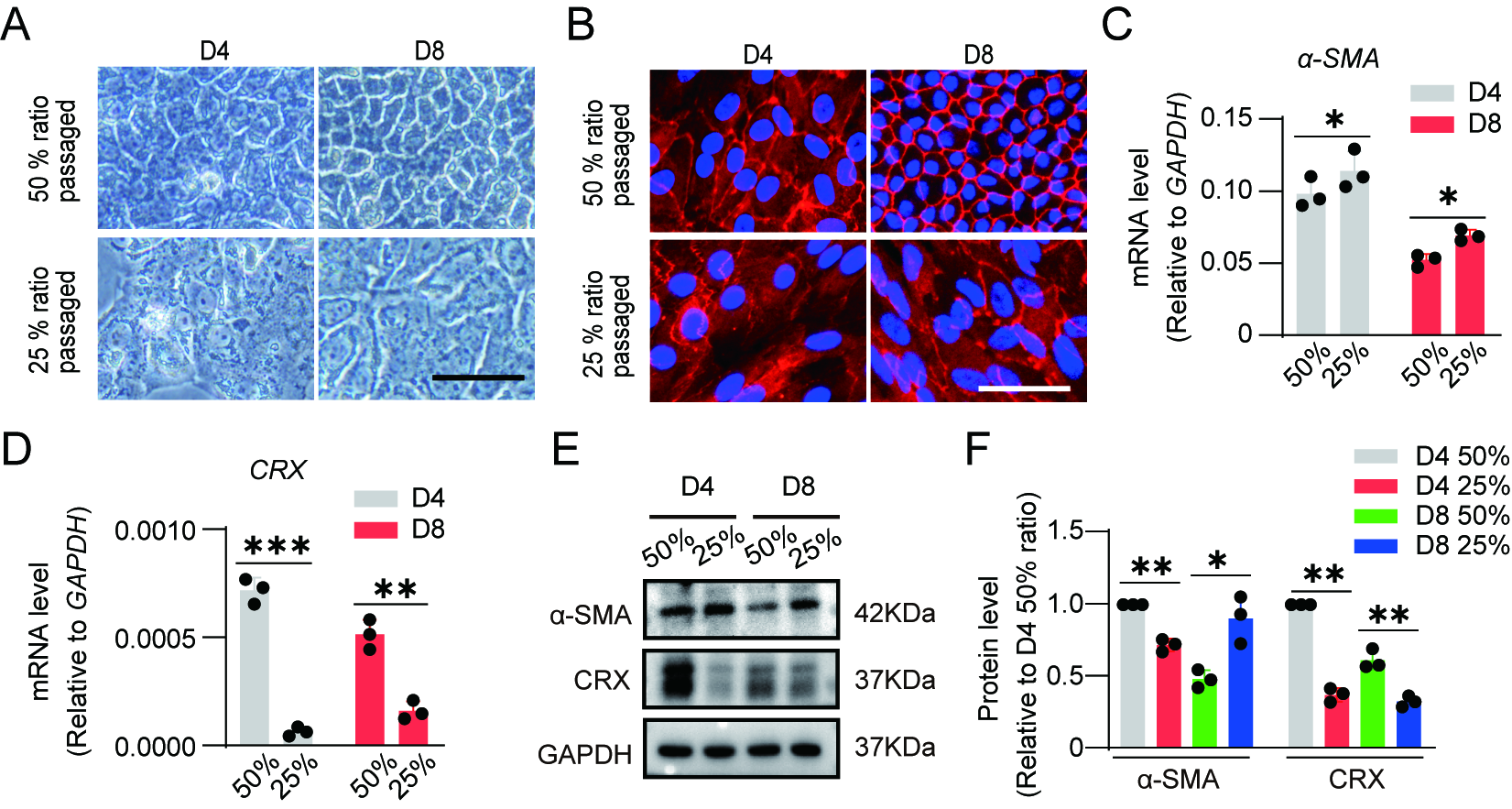

Supplement: Supplementary file 7 — Supplementary figure 6 [file 41419_2025_8352_MOESM7_ESM.tif]

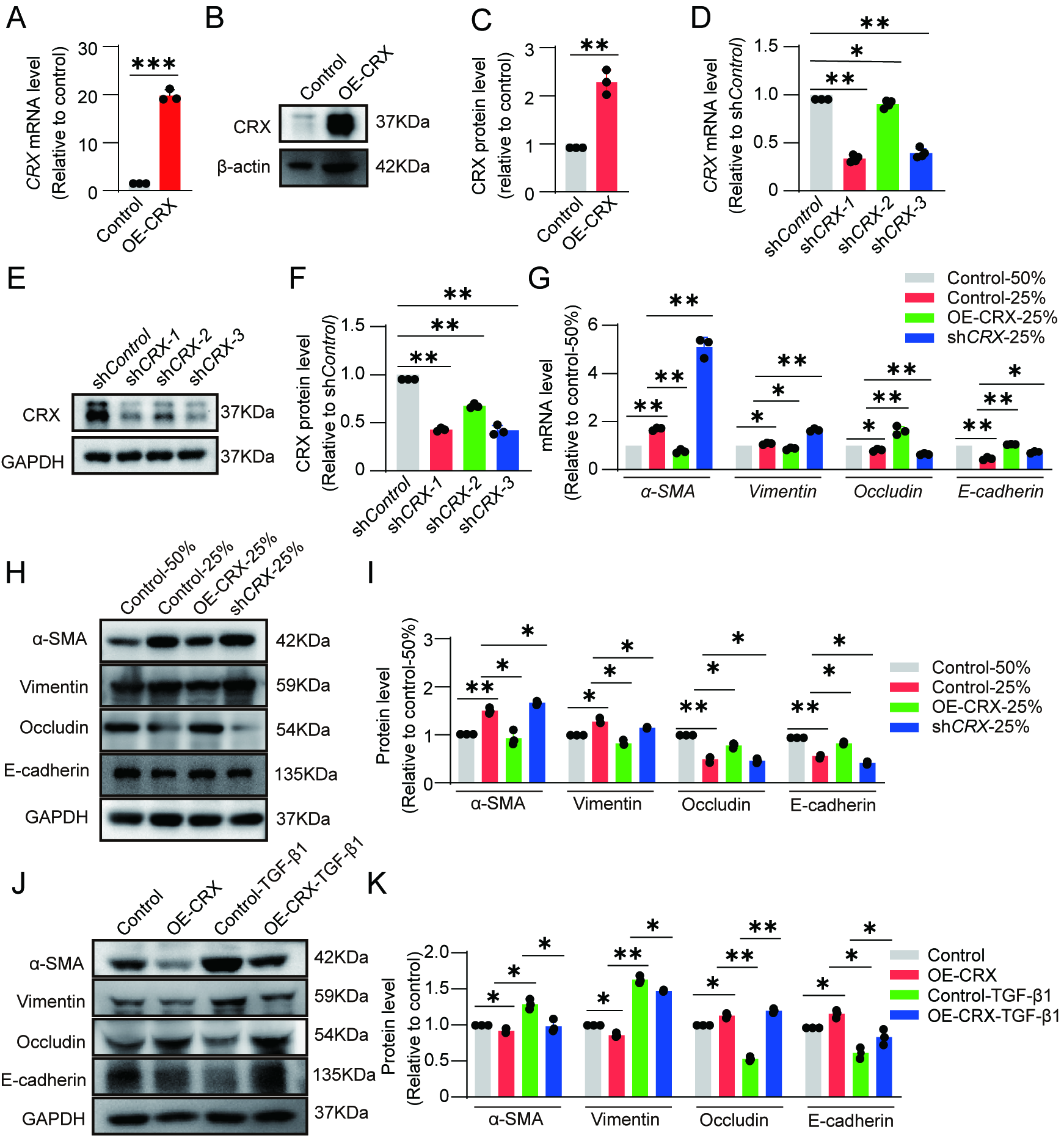

Supplement: Supplementary file 8 — Supplementary Figure 7 [file 41419_2025_8352_MOESM8_ESM.tif]

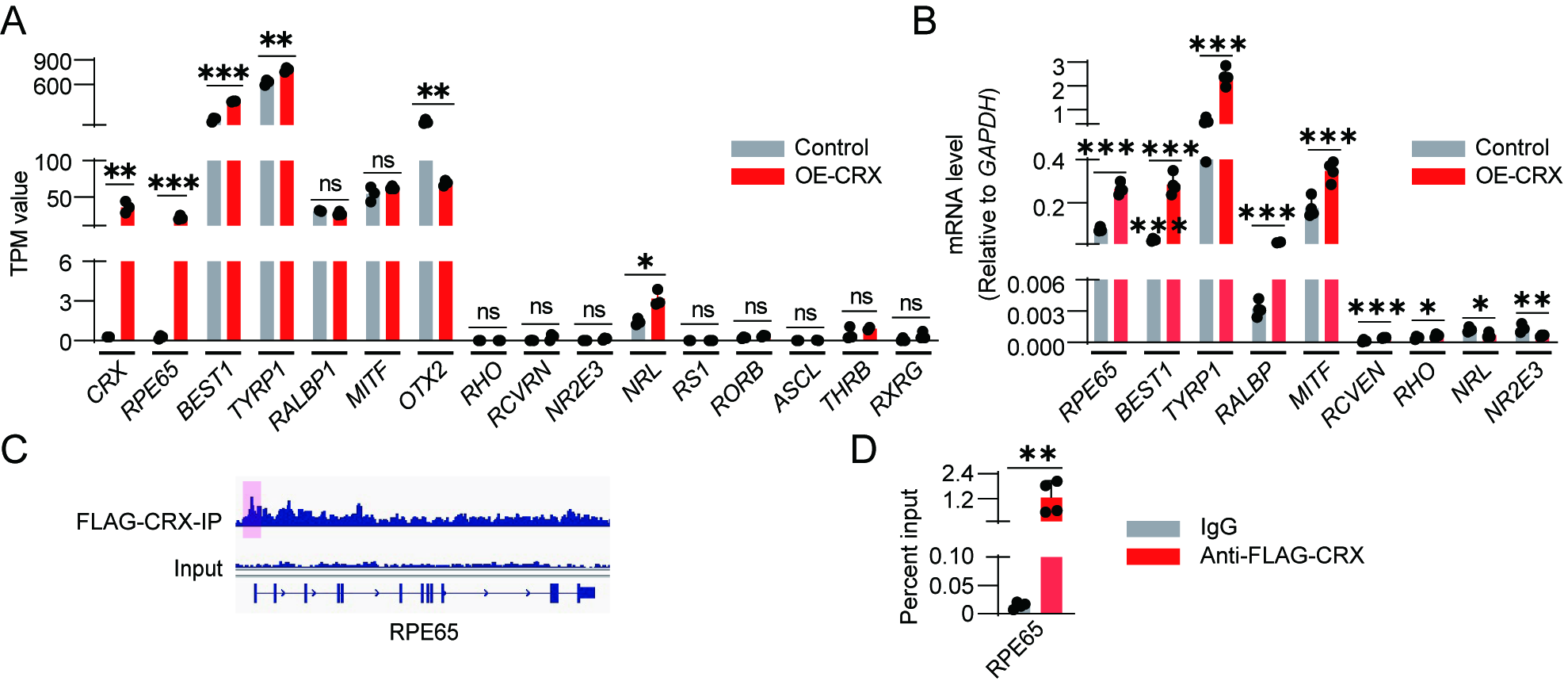

Supplement: Supplementary file 9 — Supplementary Figure 8 [file 41419_2025_8352_MOESM9_ESM.tif]

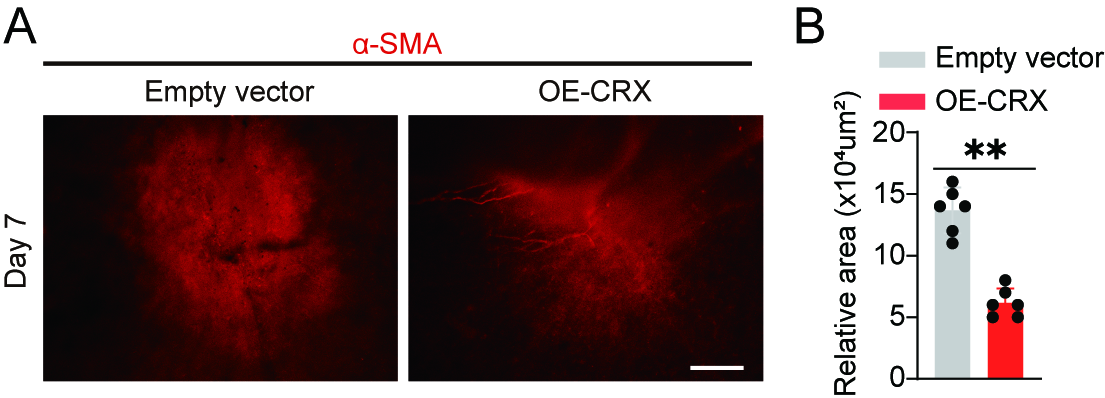

Supplement: Supplementary file 11 — Supplementary Figure 10 [file 41419_2025_8352_MOESM11_ESM.tif]
