# Supplementary material for: CRX is an intrinsic suppressor of epithelial‒mesenchymal transition in retinal pigment epithelial cells: a promising therapeutic avenue for subretinal fibrosis
Source: Cell Death Dis. 2025 Dec 31;17(1):156. doi: 10.1038/s41419-025-08352-y (PMC12859066; doi:10.1038/s41419-025-08352-y)
Supplement: Supplementary file 12 — Supplementary Tables [file 41419_2025_8352_MOESM12_ESM.docx]

**Supplementary table 1. Antibodies for Immunostaining and Western blotting.**

| **Antibody name** | **Manufacturer** | **Catalog #** | **Resource** |
| --- | --- | --- | --- |
| Anti-YAP1 polyclonal antibody | proteintech | 13584-1-AP | Mouse |
| Anti-CRX polyclonal antibody | proteintech | 12047-1-AP | Rabbit |
| Anti-phospho-YAP1 (Ser397) polyclonal antibody | proteintech | 29018-1-AP | Rabbit |
| Anti-β-catenin polyclonal antibody | proteintech | 51067-2-AP | Rabbit |
| TCF1/TCF7 (C63D9) rabbit mAb | CST | 2203S | Rabbit |
| Anti-PPP2R2B antibody [EPR10961] | abcam | ab157461 | Rabbit |
| Anti-α-smooth muscle actin antibody[1A4] | abcam | ab7817 | Mouse |
| Anti- ZO-1 Antibody (ZO1-1A12) | Thermofisher | 33-9100 | Mouse |
| Anti-RPE65 antibody (401.8B11.3D9) | Novus Biologicals | NB100-355 | Mouse |
| Anti-collagen type I polyclonal antibody | proteintech | 14695-1-AP | Rabbit |
| Anti-GAPDH monoclonal antibody | proteintech | 60004-1-Ig | Mouse |
| Anti-β-actin monoclonal antibody | proteintech | 66009-1-Ig | Mouse |
| Anti-occludin antibody | proteintech | 27260-1-AP | Rabbit |
| Anti-vimentin antibody | proteintech | 10366-1-AP | Rabbit |
| Anti-E-cadherin (4A2) antibody | CST | 14472s | Mouse |

**Supplementary table 2. Primers for qRT-PCR.**

| **Primer name** | **Primer sequence** | | **Species** |
| --- | --- | --- | --- |
| *Gapdh* | forward | ACATCGCTCAGACACCATG | human |
|  | reverse | TGTAGTTGAGGTCAATGAAGGG |  |
| *Crx* | forward | AGAACCGGAGGGCTAAAT | human |
|  | reverse | ACATCTGTGGAGGGTCTT |  |
| *Tcf7* | forward | TTAAGGAGAGCGCTGCCATC | human |
|  | reverse | CCAGTTTGTCTCTGTGGTGGAT |  |
| *a-SMA* | forward | GGGACATCAAGGAGAAACTG | human |
|  | reverse | CAGGCAACTCGTAACTCTTC |  |
| *Vimentin* | forward | CTGAACCTGAGGGAAACTAATC | human |
|  | reverse | CGTTGATAACCTGTCCATCTC |  |
| *Occludin* | forward | CCCATCTGACTATGTGGAAAG | human |
|  | reverse | GAACCGGCGTGGATTTAT |  |
| *E-cadherin* | forward | CCCTTCACAGCAGAACTAAC | human |
|  | reverse | CACCTCTAAGGCCATCTTTG |  |
| *Tgfbr3* | forward | CCGGATGGCGTAGTTTTGCC | human |
|  | reverse | GTCACTTCAGCCTGCTCAGA |  |
| *Sema4a* | forward | CGGGATGGGGTTGAGAATGG | human |
|  | reverse | CGGTGTCTGGATTCTAGGGC |  |
| *Ppp2r2b* | forward | TATTCCCGTGAAAAGGGCCAG | human |
|  | reverse | AGTCTGCAAGTACCACGTCC |  |
| *Ppm1h* | forward | CGCCGGAGTAATATGCTCACT | human |
|  | reverse | TCACAGCTGGCTTGGTCTTC |  |
| *Rai2* | forward | ACATGACTGACTCCCCTCCT | human |
|  | reverse | TTCCCTCCACCAGGTCAGT |  |
| *Usp18* | forward | CATGGCGCTTGAGAGATTCC | human |
|  | reverse | CAACCAGGCCATGAGGGTAG |  |
| *Foxs1* | forward | CACCCTCAGTGGCATCTACC | human |
|  | reverse | CGTGCTCAAACATGTCGTGG |  |
| *Gdf6* | forward | CGATCTCTCGCACACTCCTC | human |
|  | reverse | CACACGTCGAAGACTTCCCA |  |
| *Cd44* | forward | AGTCACAGACCTGCCCAATG | human |
|  | reverse | TTGCCTCTTGGTTGCTGTCT |  |
| *Fn1* | forward | GGAGATTCATGGGAGAAGTATG | human |
|  | reverse | GACCACTTGAGCTTGGATAG |  |
| *Tyrp1* | forward | CTCTCTCTGGGCTGTATCTT | human |
|  | reverse | GGCAACACATACCACTTCTC |  |
| *Rpe65* | forward | TTTCTCACCCAGATGCCTTG | human |
|  | reverse | TTAATCTCCACTTCAGCCCG |  |
| *Ralbp1* | forward | TACTTCACCACGACCTACA | human |
|  | reverse | CCTGGTAGAAACCAGAAAGG |  |
| *Best1* | forward | TCATCCCCATTTCCTTCGTG | human |
|  | reverse | TTGCTCGTCCTTGCCTTC |  |
| *Mitf* | forward | AGGAAATCTTGGGCTTGATGG | human |
|  | reverse | TGTTGGGAAGGTTGGCTG |  |
| *Rhodopsin* | forward | GAGTCTTTTGTCATCTACATGTTCG | human |
|  | reverse | CTCCTTCTCTGCCTTCTGTG |  |
| *Rcvrn* | forward | CTGGGTAAGCAGTTTCCAATA | human |
|  | reverse | GTCTTTGAGGTGTGACTGATAG |  |
| *Nrl* | forward | TTTGAGGTAAAGCGGGAAC | human |
|  | reverse | TCACTGAAGGTGGGTGAA |  |
| *Nr2e3* | forward | CTCCTCTCCATACTCCTCTT | human |
|  | reverse | GAACACAGGCAGGTTCTT |  |

**Supplementary table 3. Primers for ChIP-qRT-PCR.**

| **ChIP-qRT-PCR Primer name** | **Primer sequence** | |
| --- | --- | --- |
| *#1 for CRX* | forward | GAGAGACACAAAGAGGGCCA |
|  | reverse | TCCTTTCATTTCTGTCCTTCTCCC |
| *#2 for CRX* | forward | ACACAAAGAGGGCCAGAGACC |
|  | reverse | TCCTTTCATTTCTGTCCTTCTCCCT |
| *#3 for CRX* | forward | CAACAGAGACAGGGACGGAG |
|  | reverse | GGCCTTAGGCTGTGGCATTT |
| *#4 for CRX* | forward | CAGAGAGGCCACAGACAAGAC |
|  | reverse | AGTGCCTCATTGGTCTCGGT |
| *#5 for CRX* | forward | TCTGACAGTGCTCTCCTTCCT |
|  | reverse | GTGACCATAAGCCCCTACTCC |
| *#6 for CRX* | forward | CAGCATACCGCTCTGTTGGA |
|  | reverse | GGCCATTTGTGGATCATCGC |
| *for Ppp2r2b* | forward | CTCTGGCTTGGCTGTGATCT |
|  | reverse | TCCACTGTCAGGAACGTTGG |
| *for Rpe65* | forward | CAAAGTCACACACTAGGAAATG |
|  | reverse | CAAAGTCACACACTAGGAAATG |

**Supplementary table 4. The shRNA sequences for target genes.**

| **shRNA** | **shRNA Sequence** |
| --- | --- |
| sh*CRX_*1 | CCACTATTCTGTCAACGCCTT |
| sh*CRX_*2 | GCACCTGGAAATTCACCTACA |
| sh*CRX_*3 | TGAAGATCAATCTGCCTGAGT |
| sh*TCF7_*1 | TGATGCTAGGTTCTGGTGT |
| sh*TCF7_*2 | TGTCTTGATGTGTCATCTA |
| sh*Ppp2r2b_*1 | AGGGACTACTTGACCGTCA |
| sh*Ppp2r2b_*2 | AGGTTGTCTGTAGTATTTA |
| sh*Ppp2r2b_*3 | AGGAAATGATTGGAATAGA |
| sh*Control* | GCGCGATAGCGCTAATAATTT |
