# Supplementary material for: CRX is an intrinsic suppressor of epithelial‒mesenchymal transition in retinal pigment epithelial cells: a promising therapeutic avenue for subretinal fibrosis
Source: Cell Death Dis. 2025 Dec 31;17(1):156. doi: 10.1038/s41419-025-08352-y (PMC12859066; doi:10.1038/s41419-025-08352-y)
Supplement: Supplementary file 13 — Western blotting original figures [file 41419_2025_8352_MOESM13_ESM.pdf]

Figure.2D

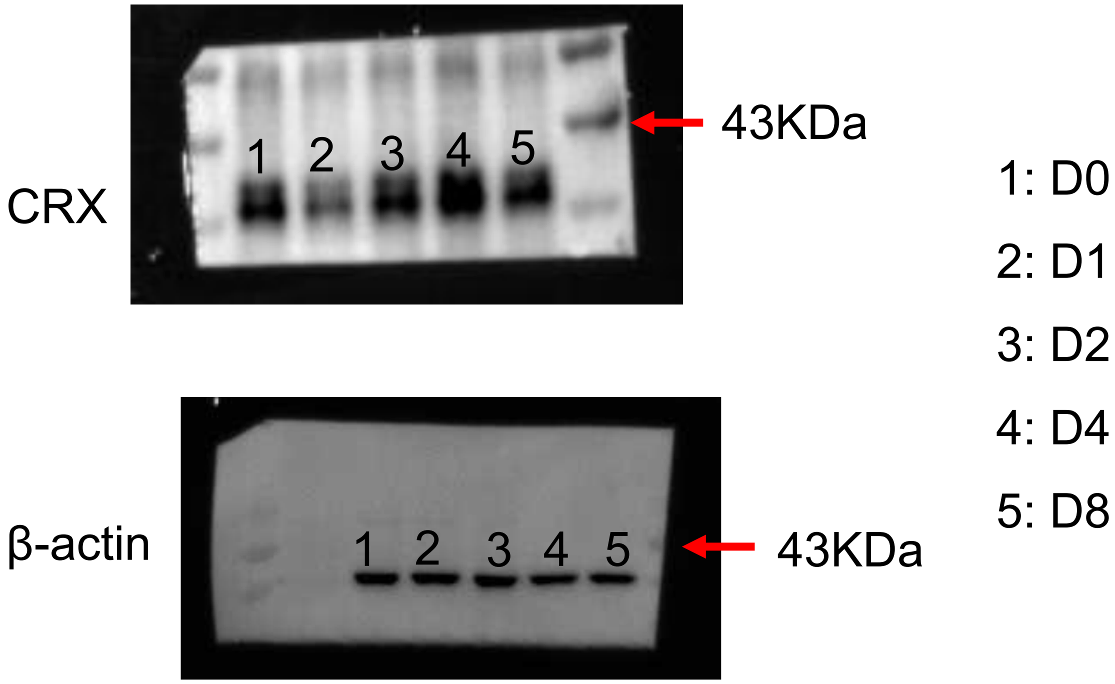

Figure.3F

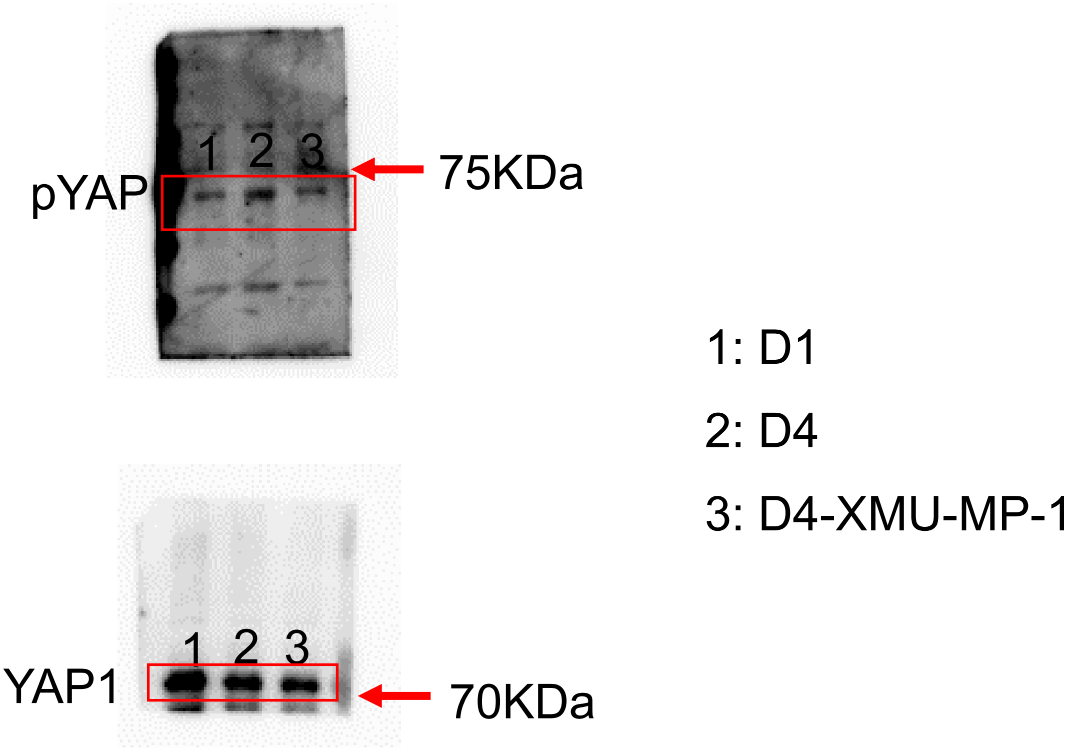

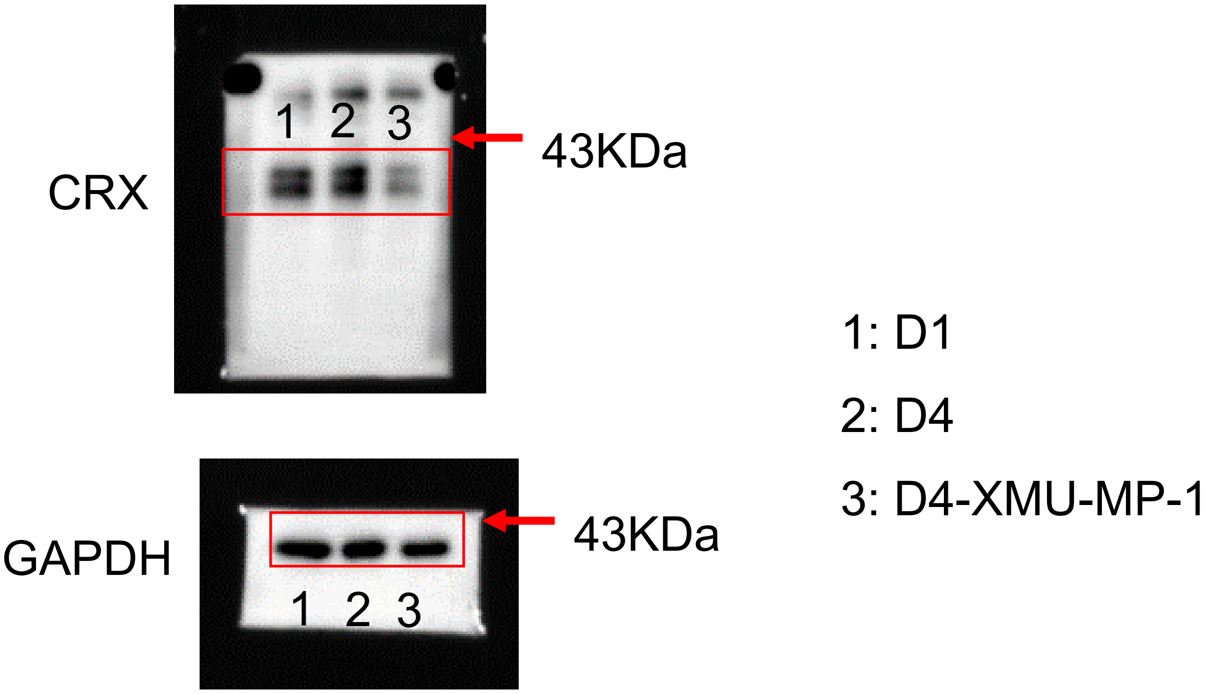
CRX
1 2 3
43KDa
GAPDH
1 2 3
43KDa
1: D1
2: D4
3: D4-XMU-MP-1

Figure.3I

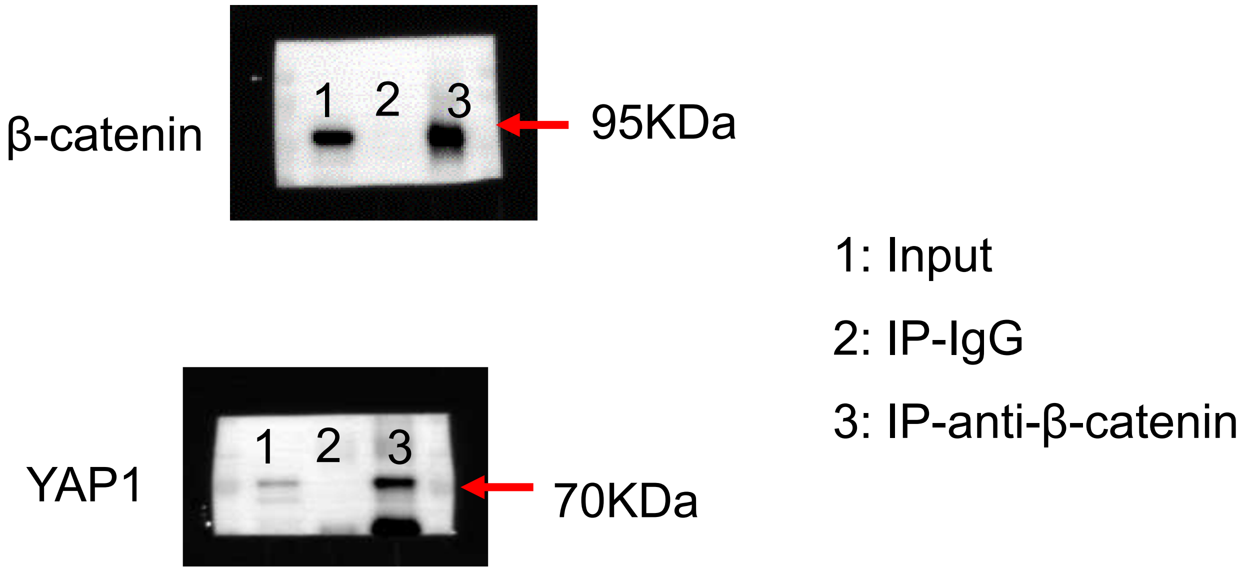
β-catenin
1 2 3
95KDa
YAP1
1 2 3
70KDa
1: Input
2: IP-IgG
3: IP-anti-β-catenin

Figure.3J

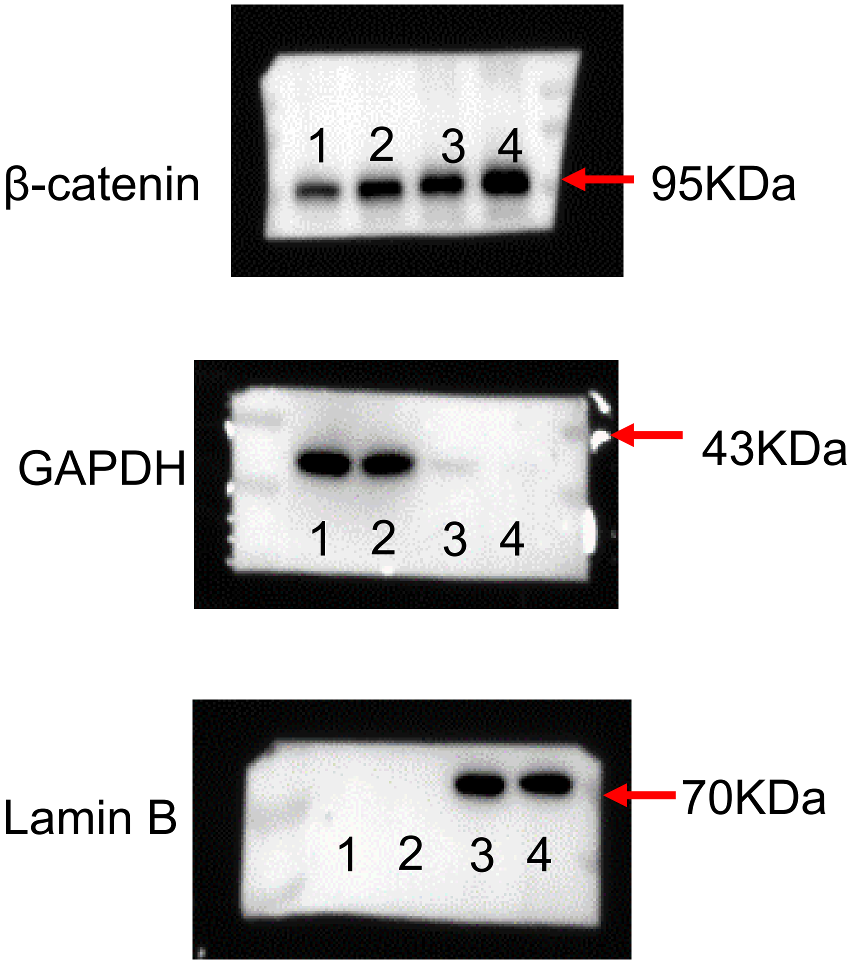

1: Control (Cytosol)

2: XMU-MP-1 (Cytosol)

3: Control (Nucleus)

4: XMU-MP-1(Nucleus)

Figure.3M

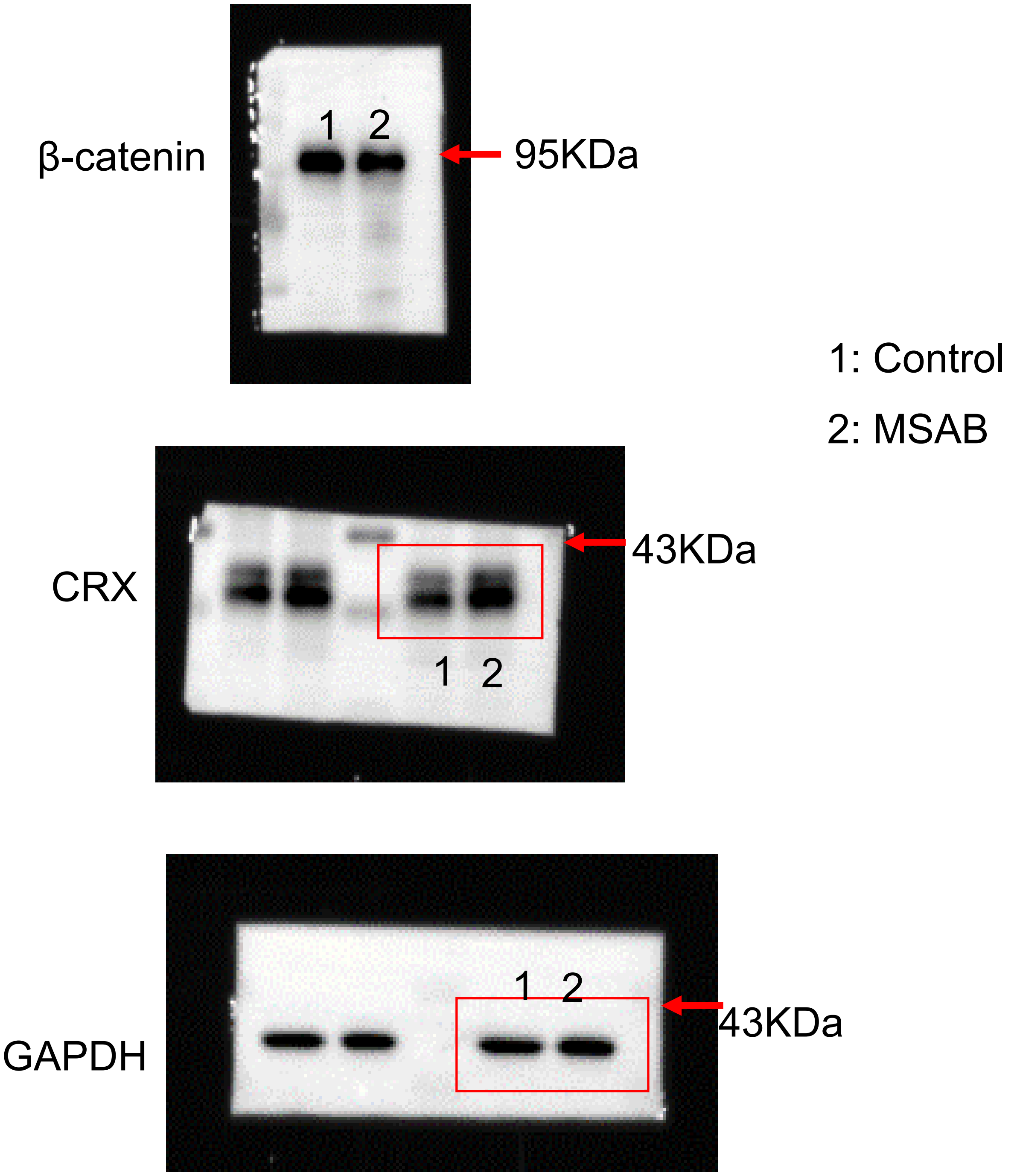
β-catenin
1
2
95KDa
CRX
43KDa
1
2
GAPDH
1
2
43KDa
1: Control
2: MSAB

Figure.3S

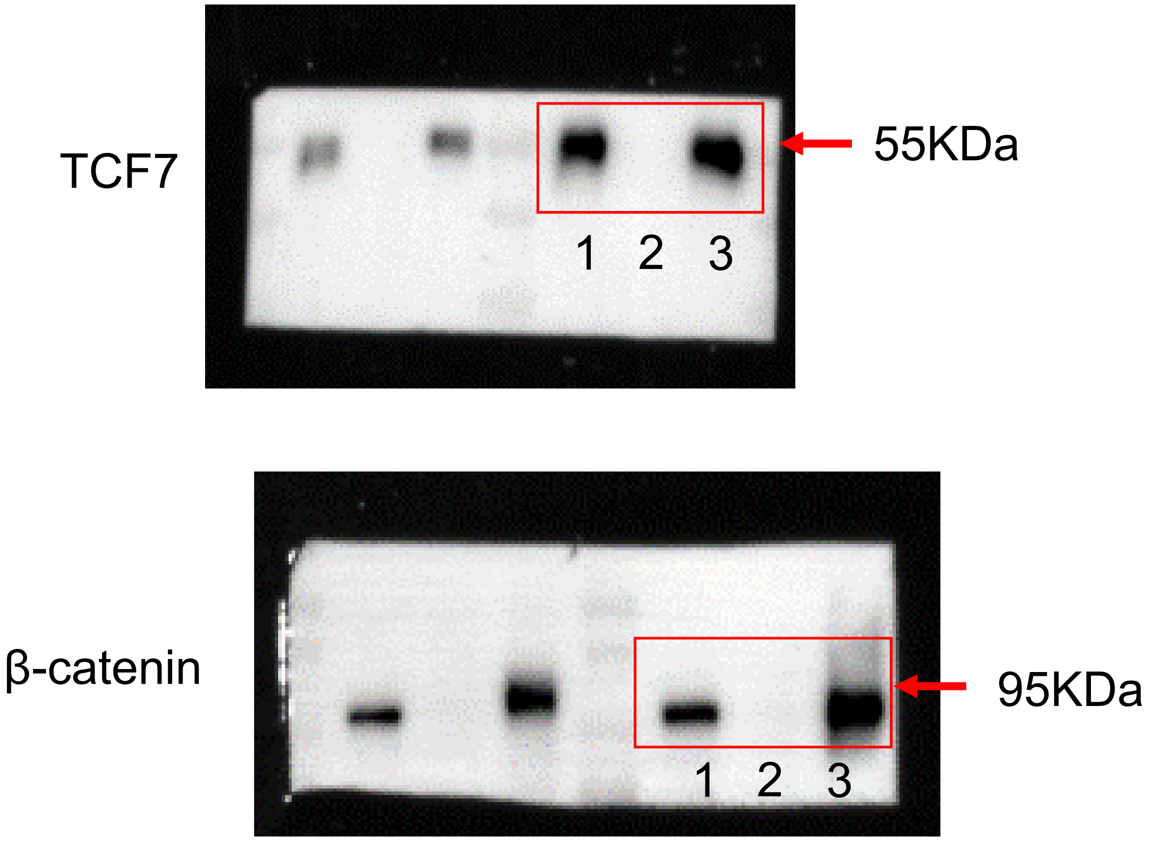

1: Input

2: IP-IgG

3: IP-anti-TCF7

Figure.3T

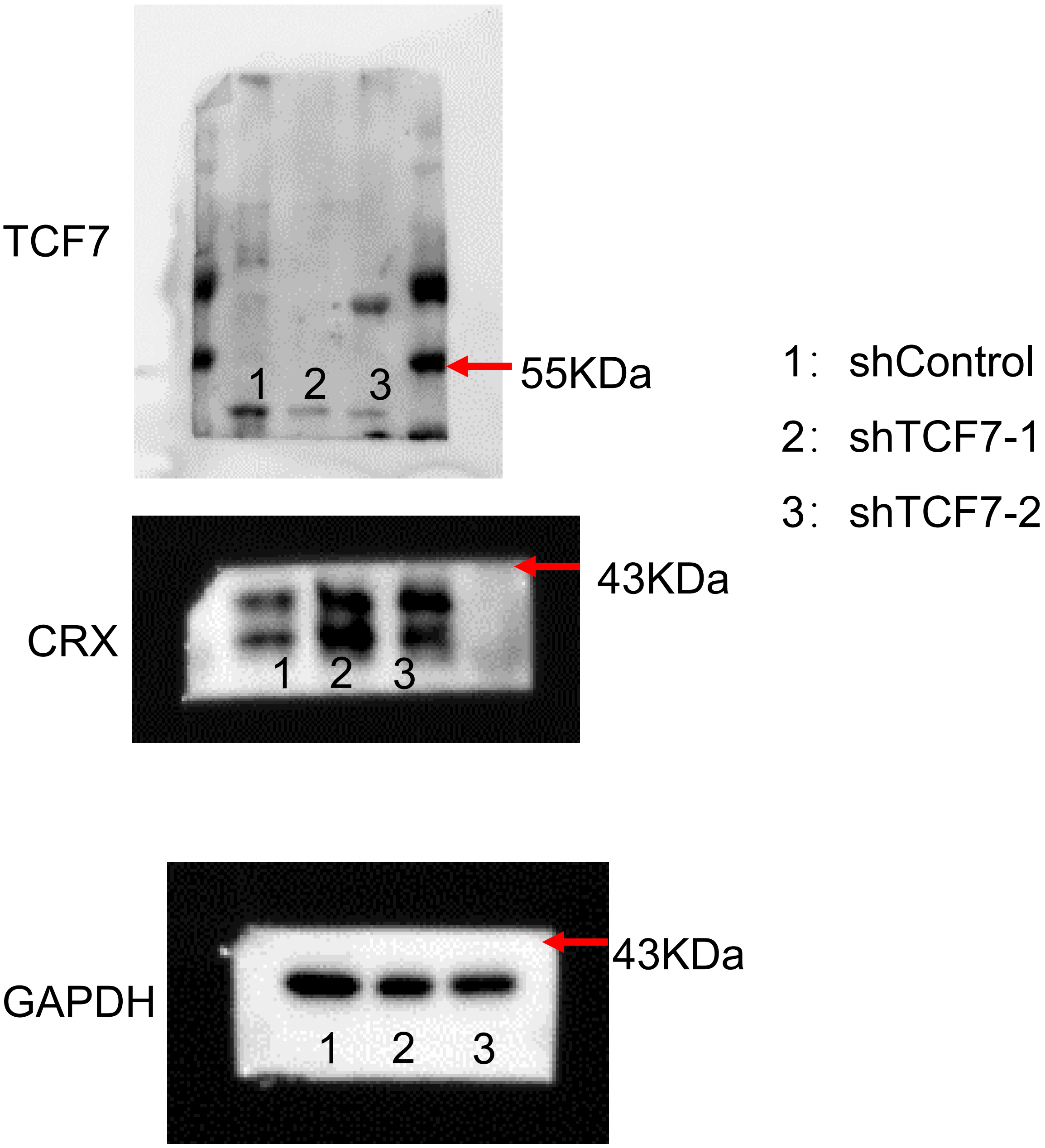

TCF7
1 2 3
55KDa
CRX
1 2 3
43KDa
GAPDH
1 2 3
43KDa
1： shControl
2： shTCF7-1
3： shTCF7-2

Figure.4B

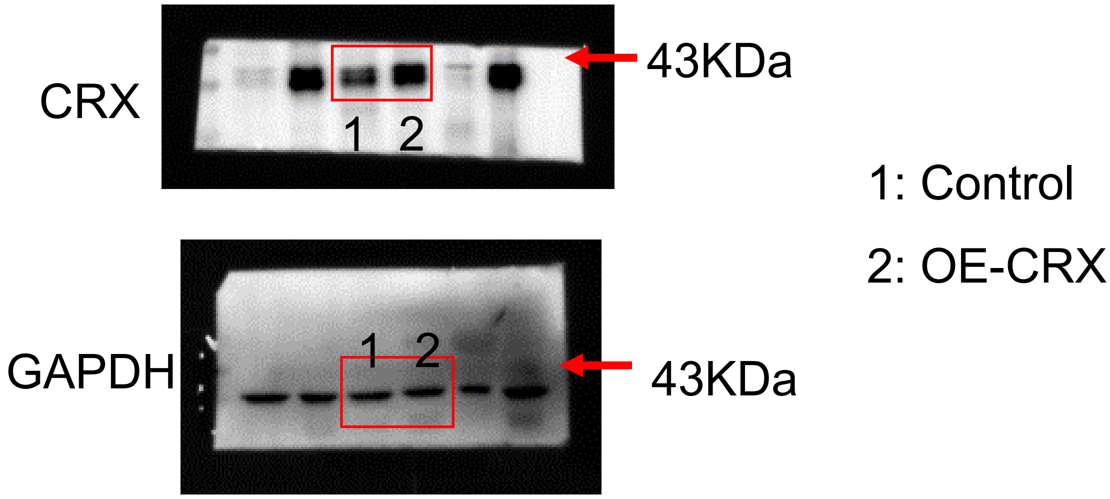

Figure.4E

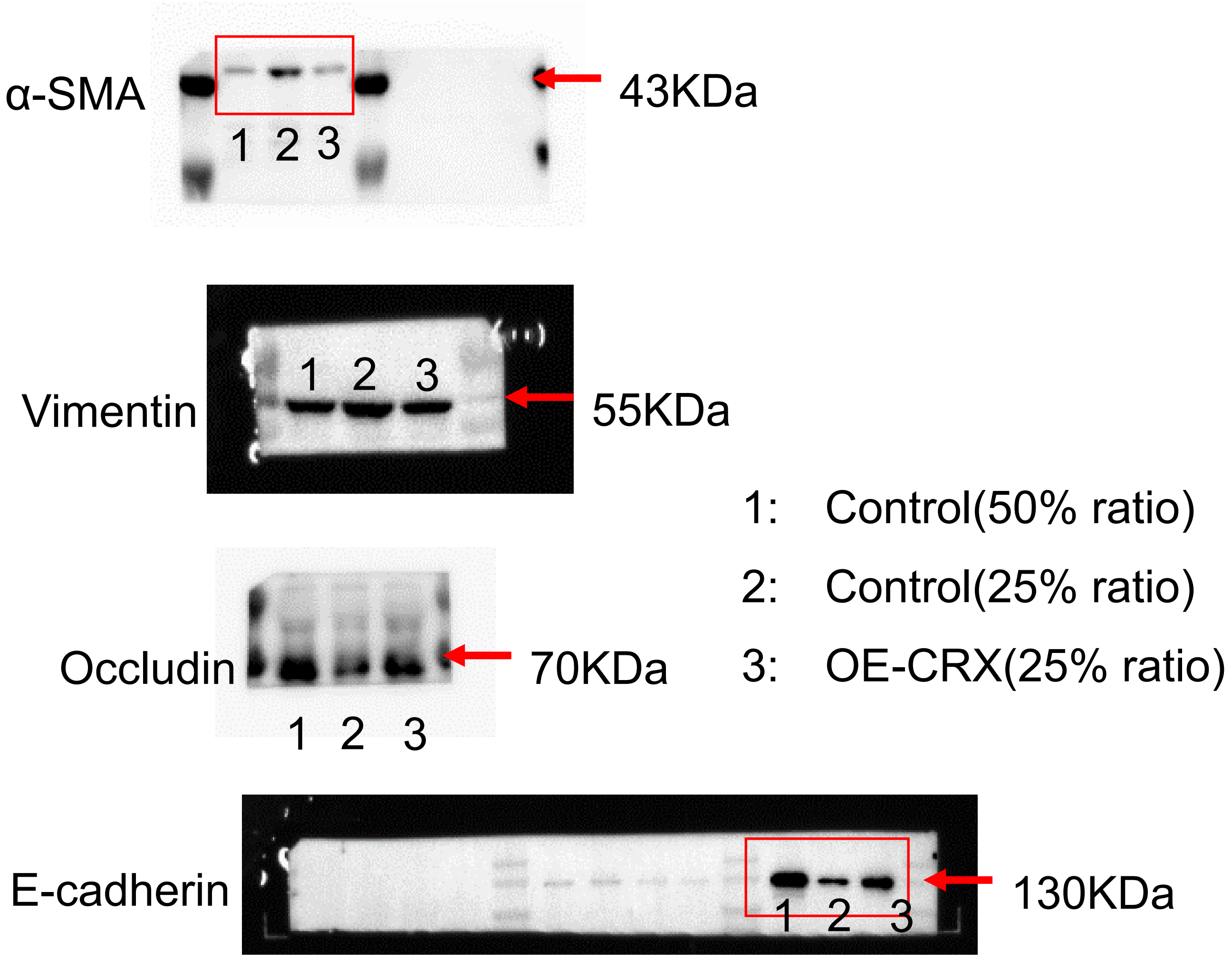

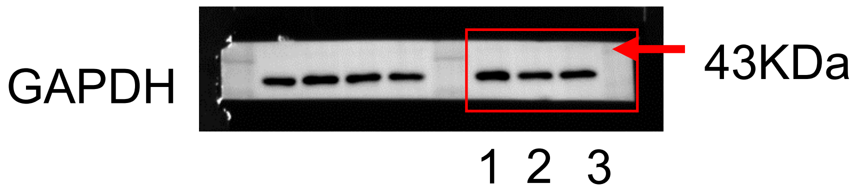

Figure.4H

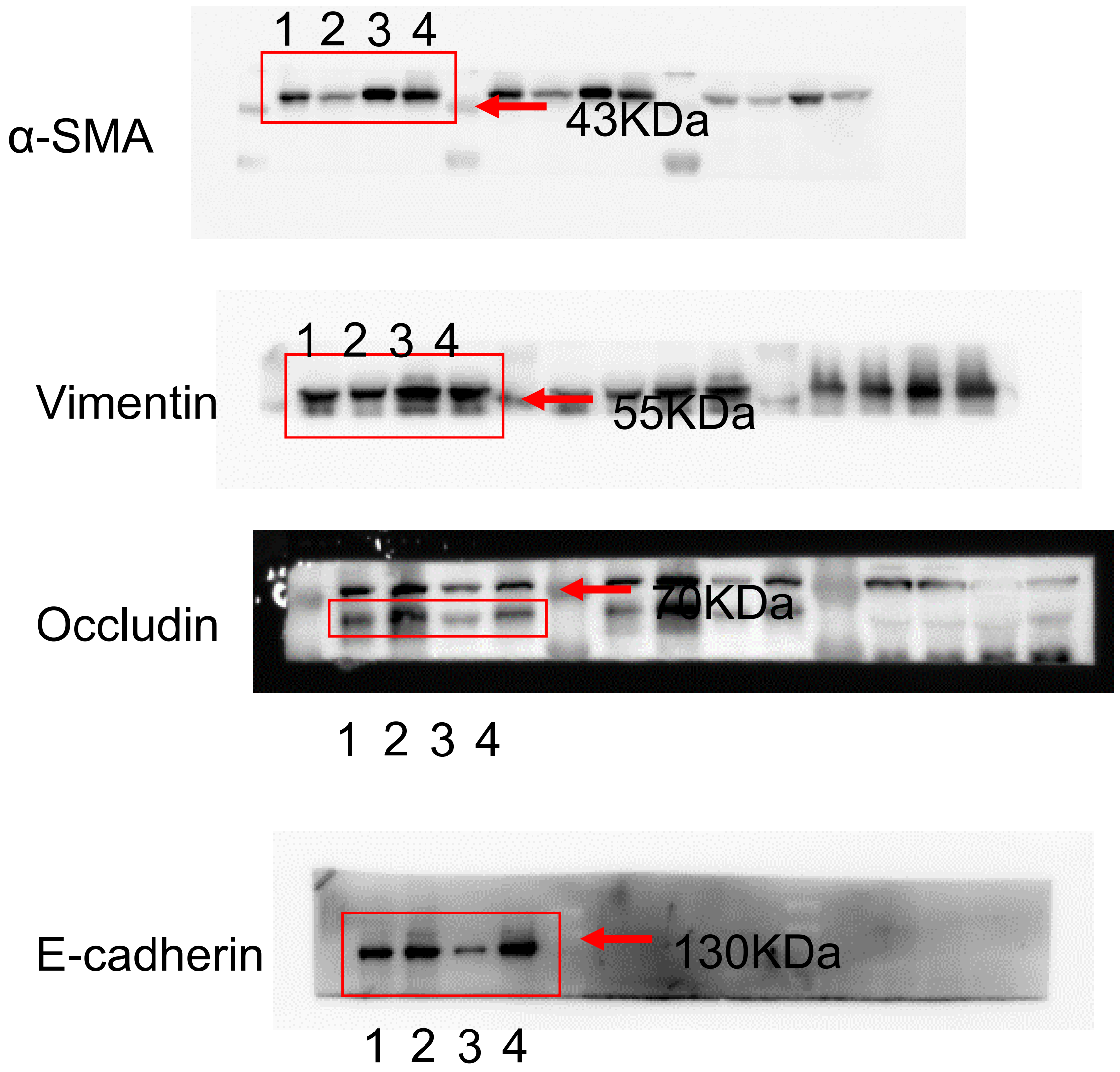

1: Control

2: OE-CRX

3: Control-TGF-β1

4: OE-CRX-TGF-β1

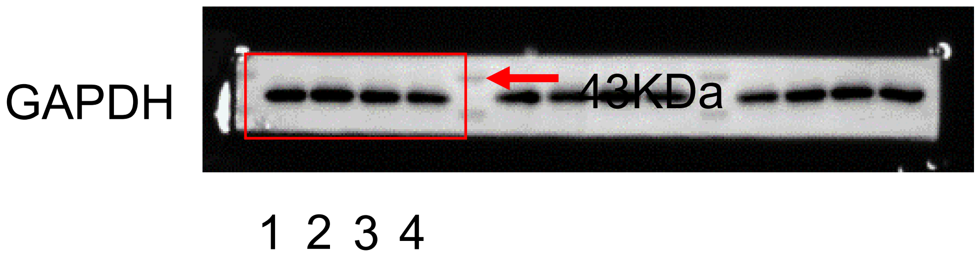

Figure.4J

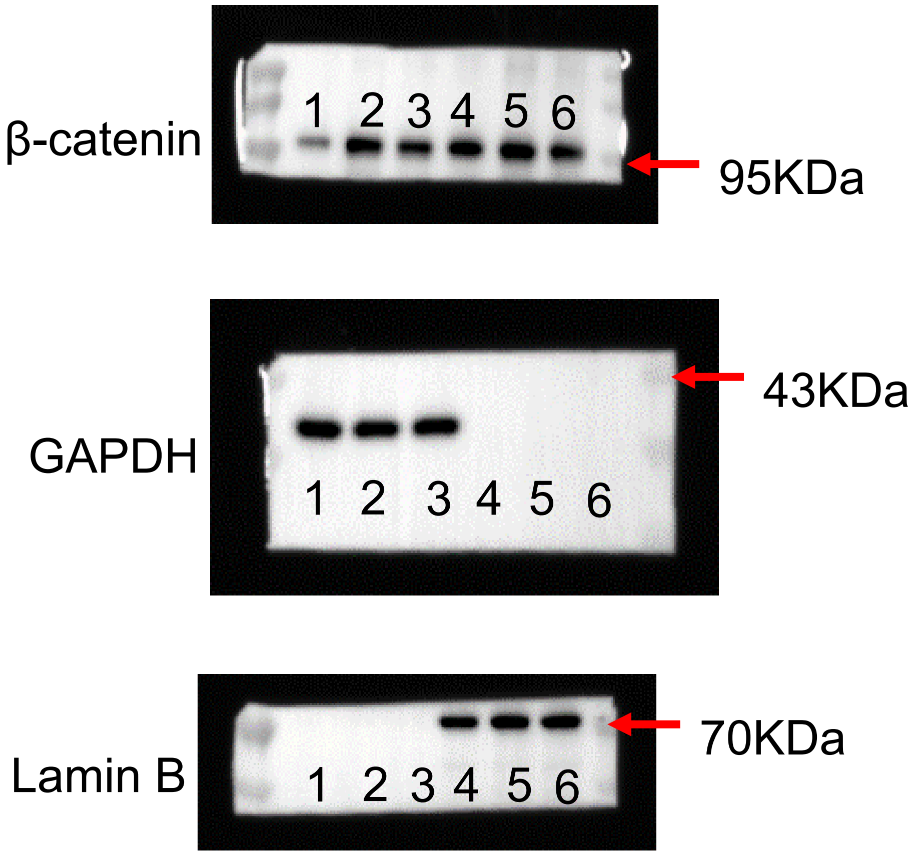

1: Control (Cytosol)

2: TGF-β1 (Cytosol)

3: TGF-β1+MSAB (Cytosol)

4: Control (Nucleus)

5: TGF-β1 (Nucleus)

6: TGF-β1+MSAB (Nucleus)

Figure.4M

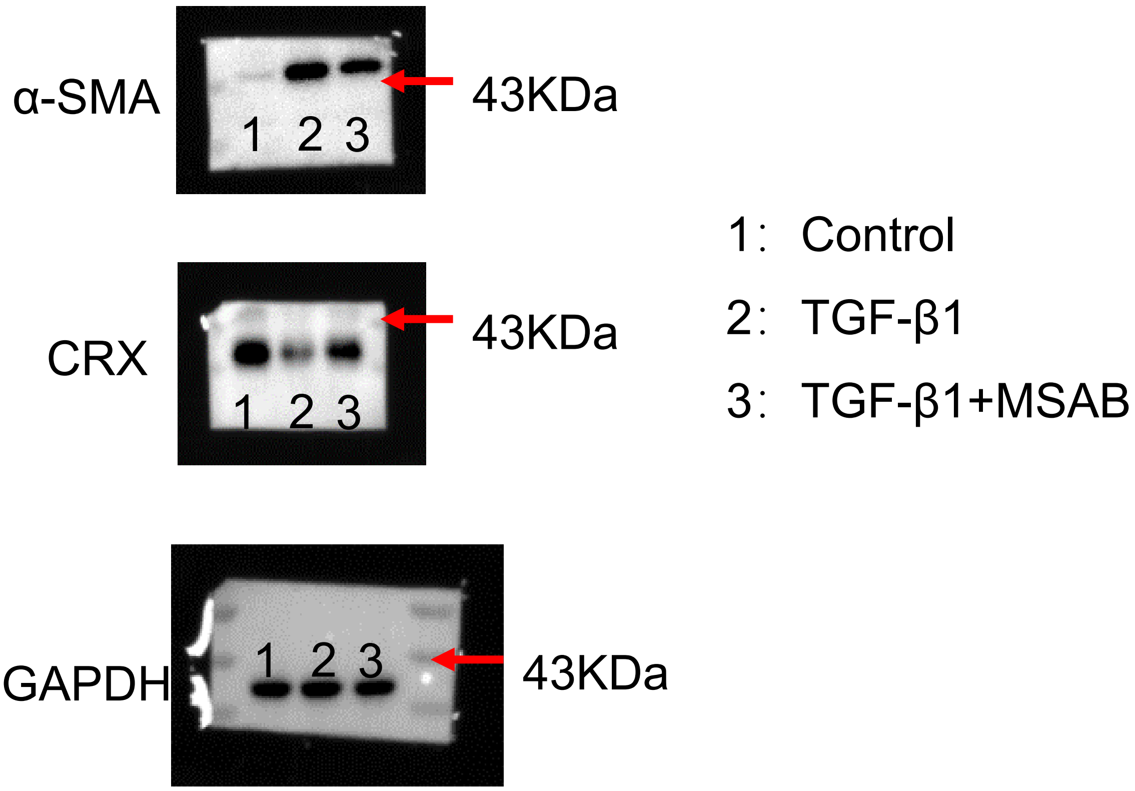

Figure.5G

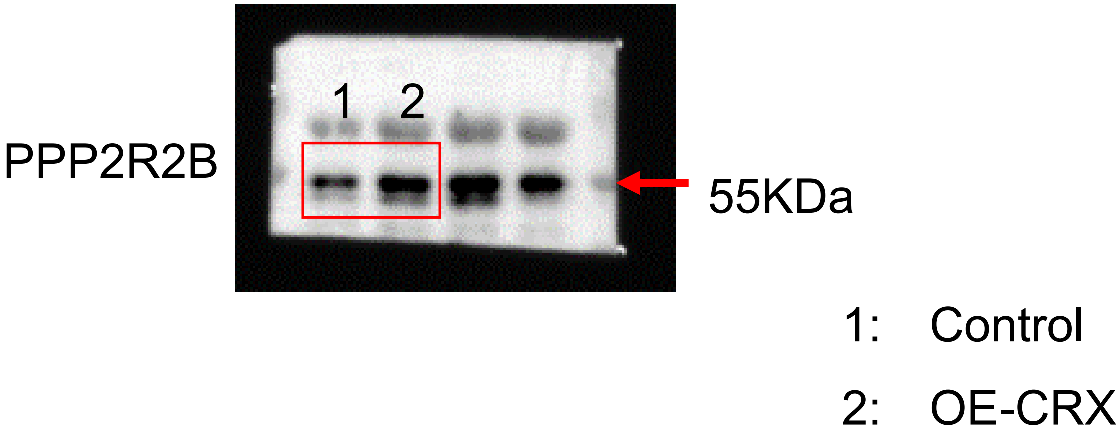

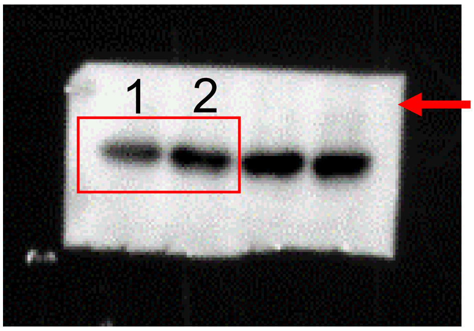

Figure.6C

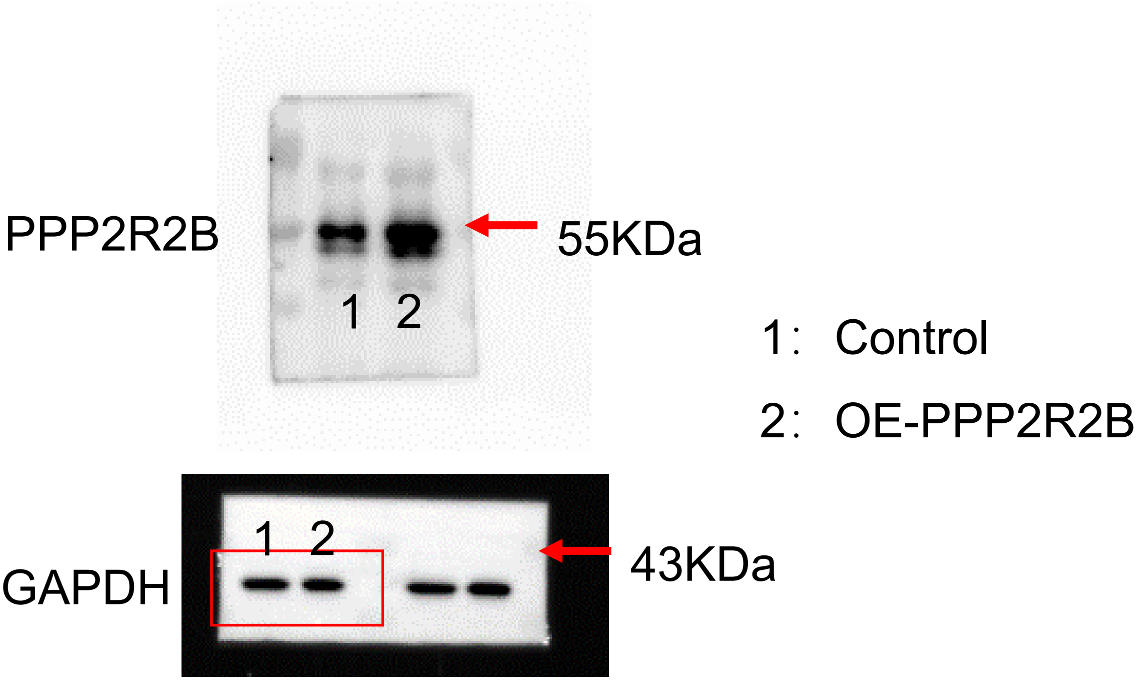

Figure.6E

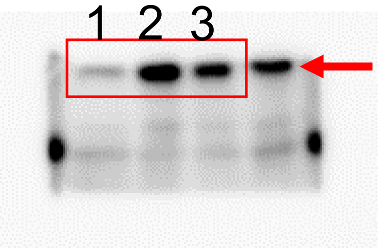

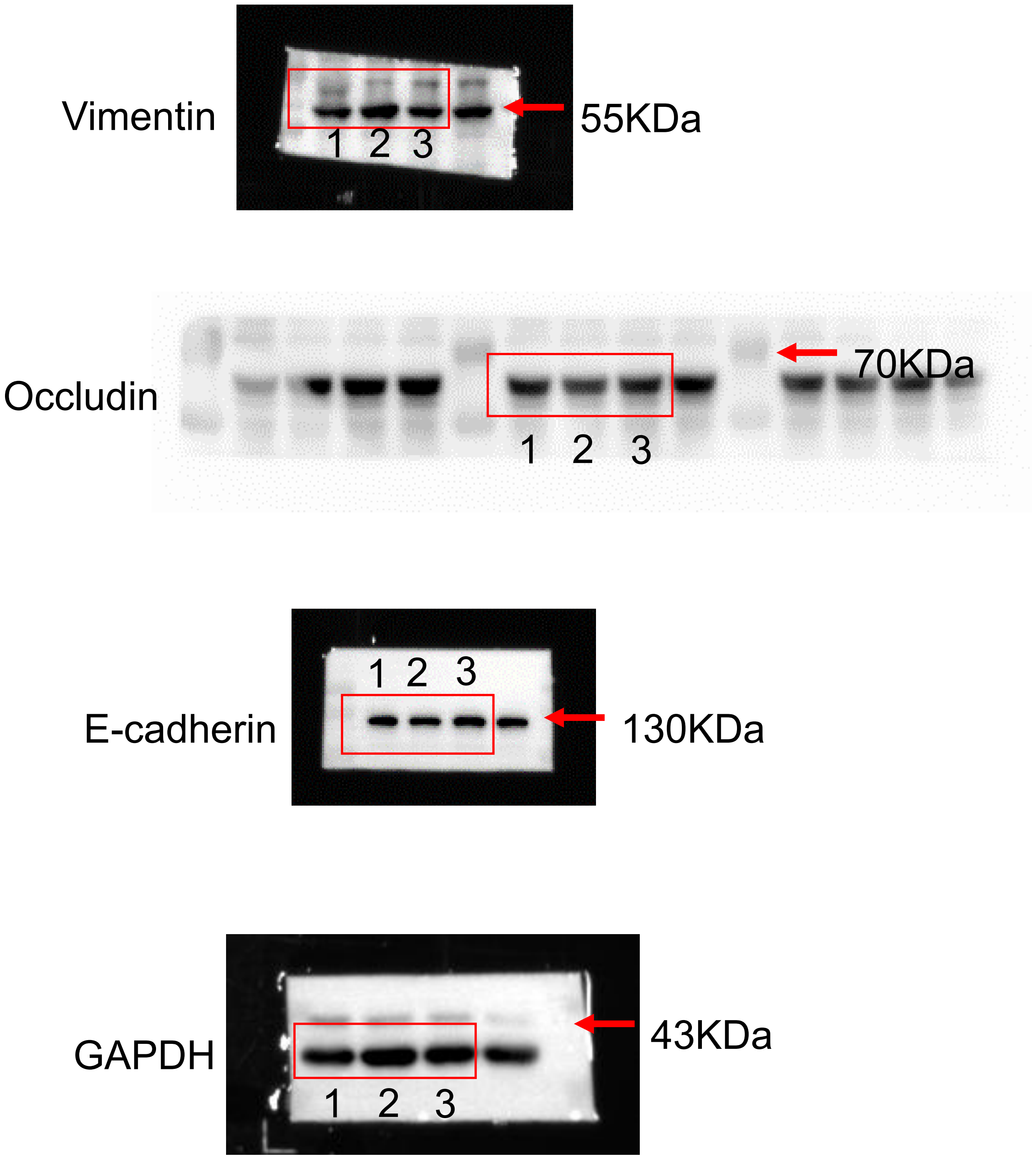

1： Control

2： Control-TGF-β1

3： OE-PPP2R2B-TGF-β1

Figure.6H

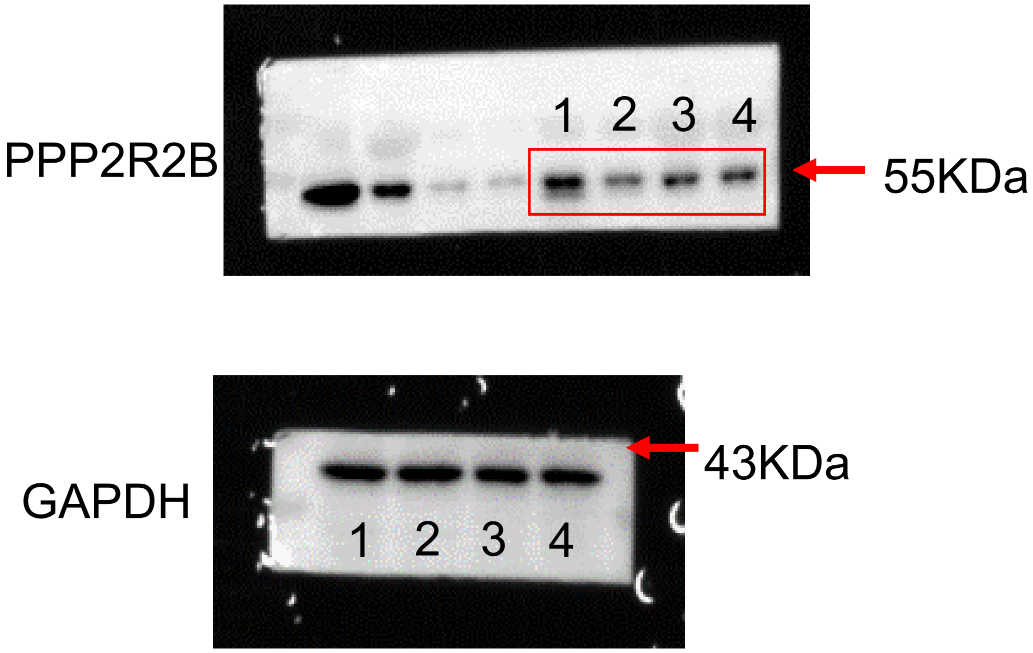

1： OE-CRX-shControl

2： OE-CRX-shPPP2R2B-1

3： OE-CRX-shPPP2R2B-2

4： OE-CRX-shPPP2R2B-3

Figure.6J

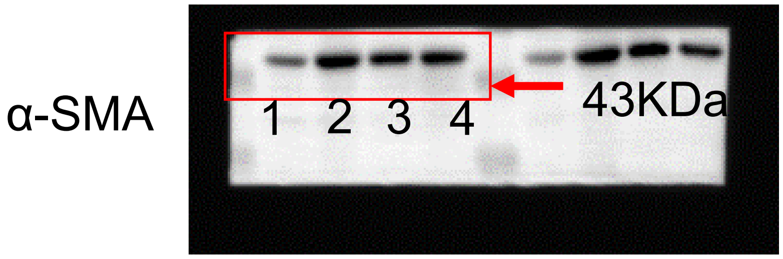

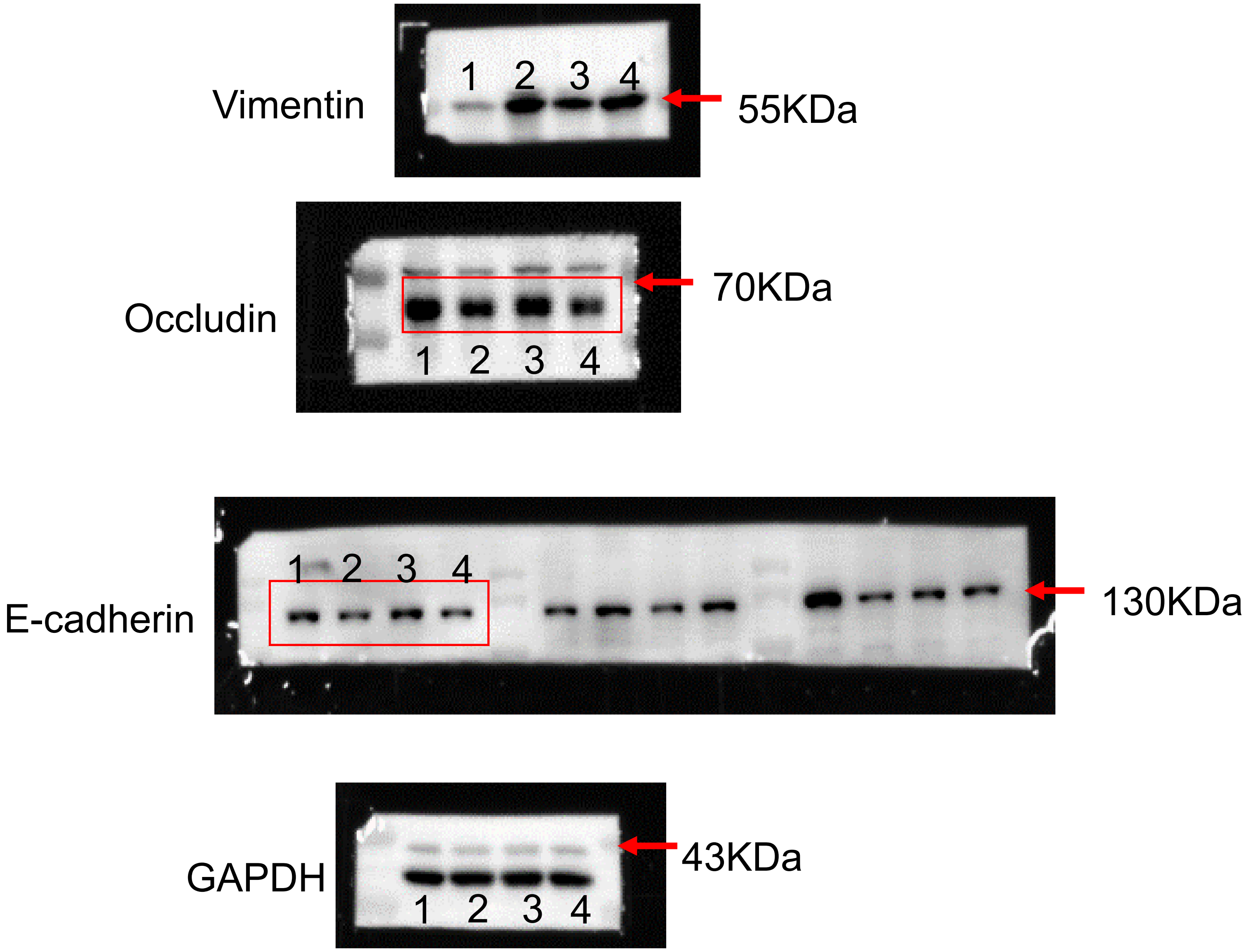

1: shControl

2: shControl-TGF-β1

3: OE-CRX-TGF-β1

4: OE-CRX-shPPP2R2B-TGFβ1

Supplementary figure.1A

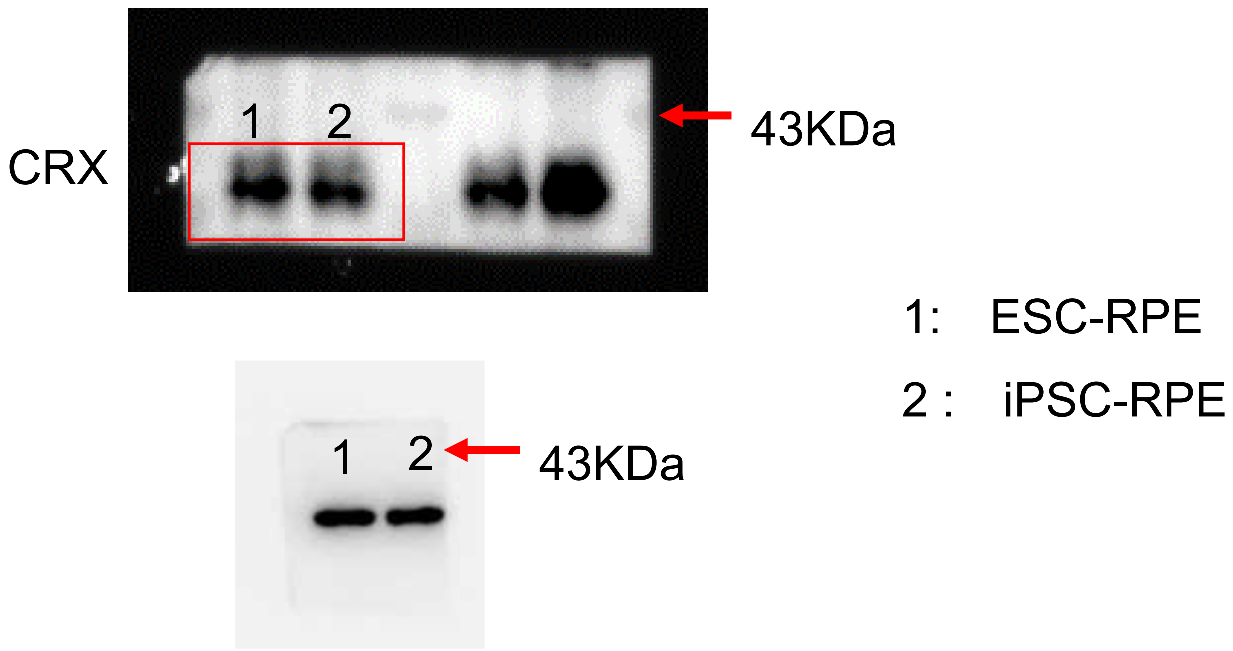

Supplementary figure.6E

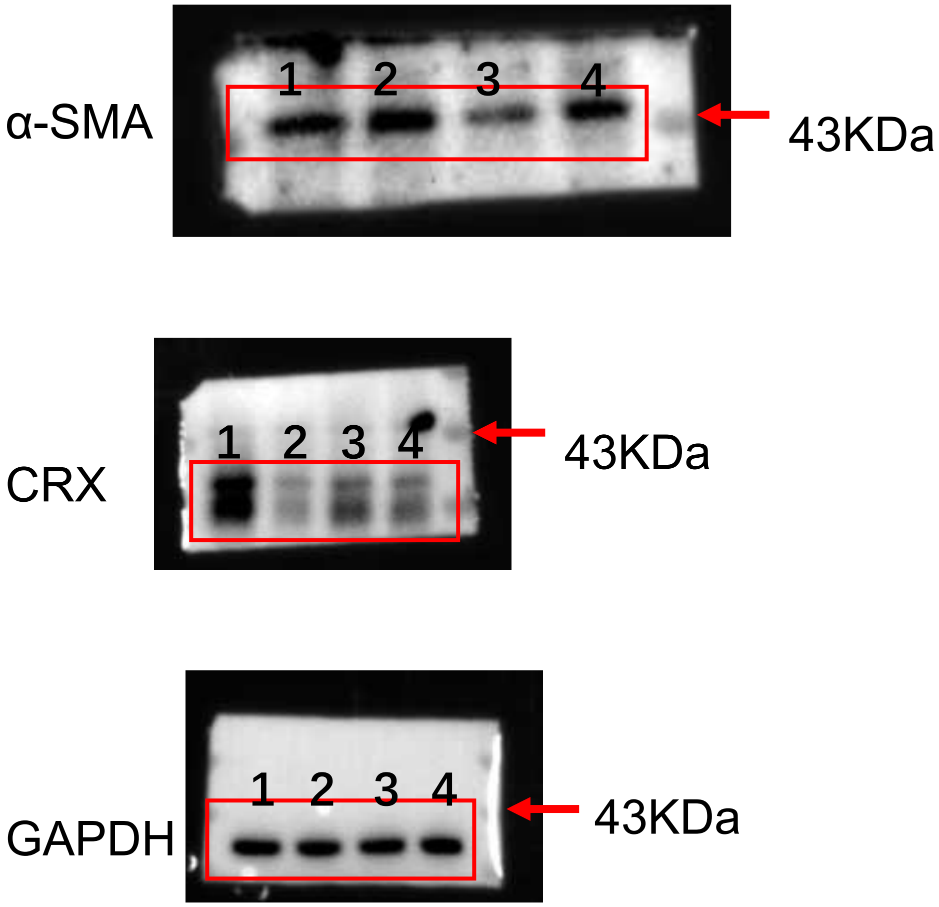

Supplementary figure.7B

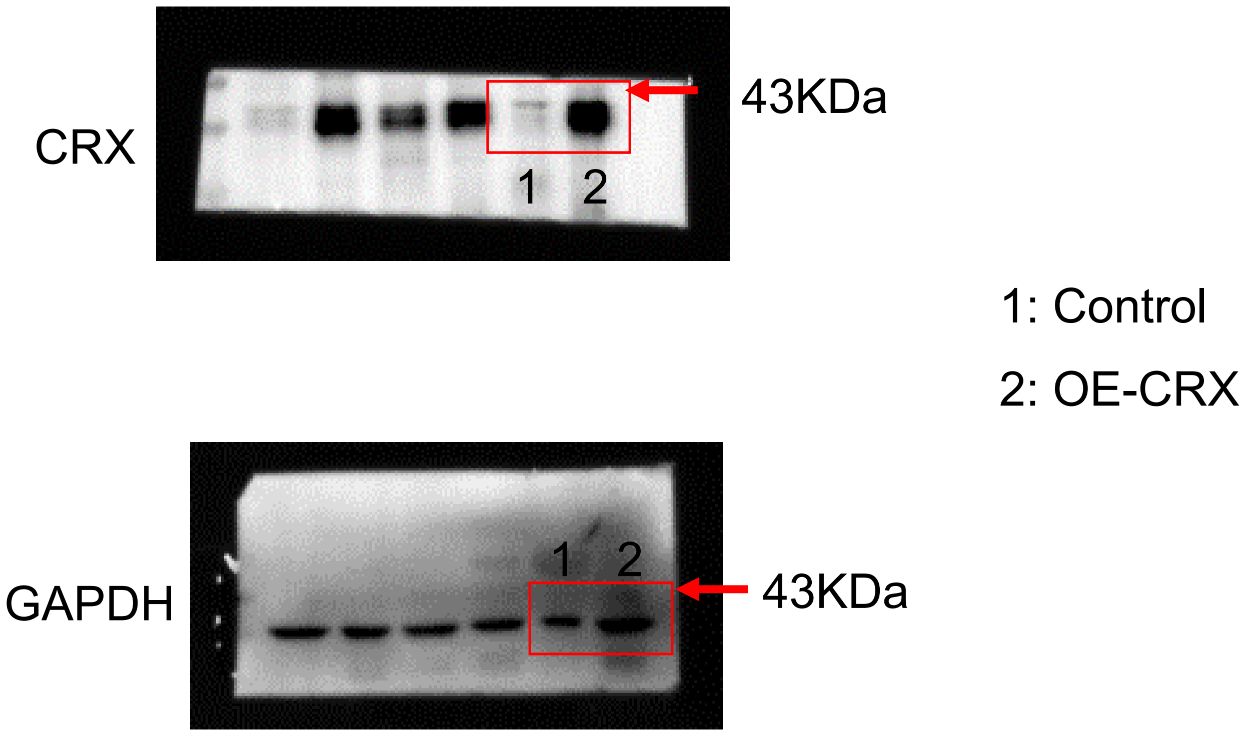

Supplementary figure.7E

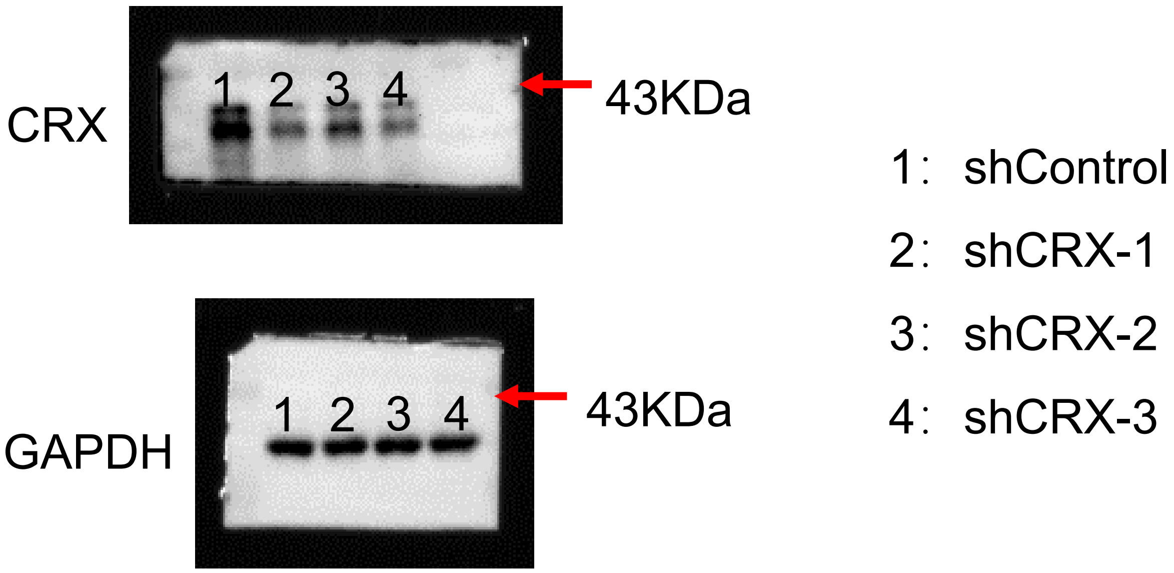

Supplementary figure.7H

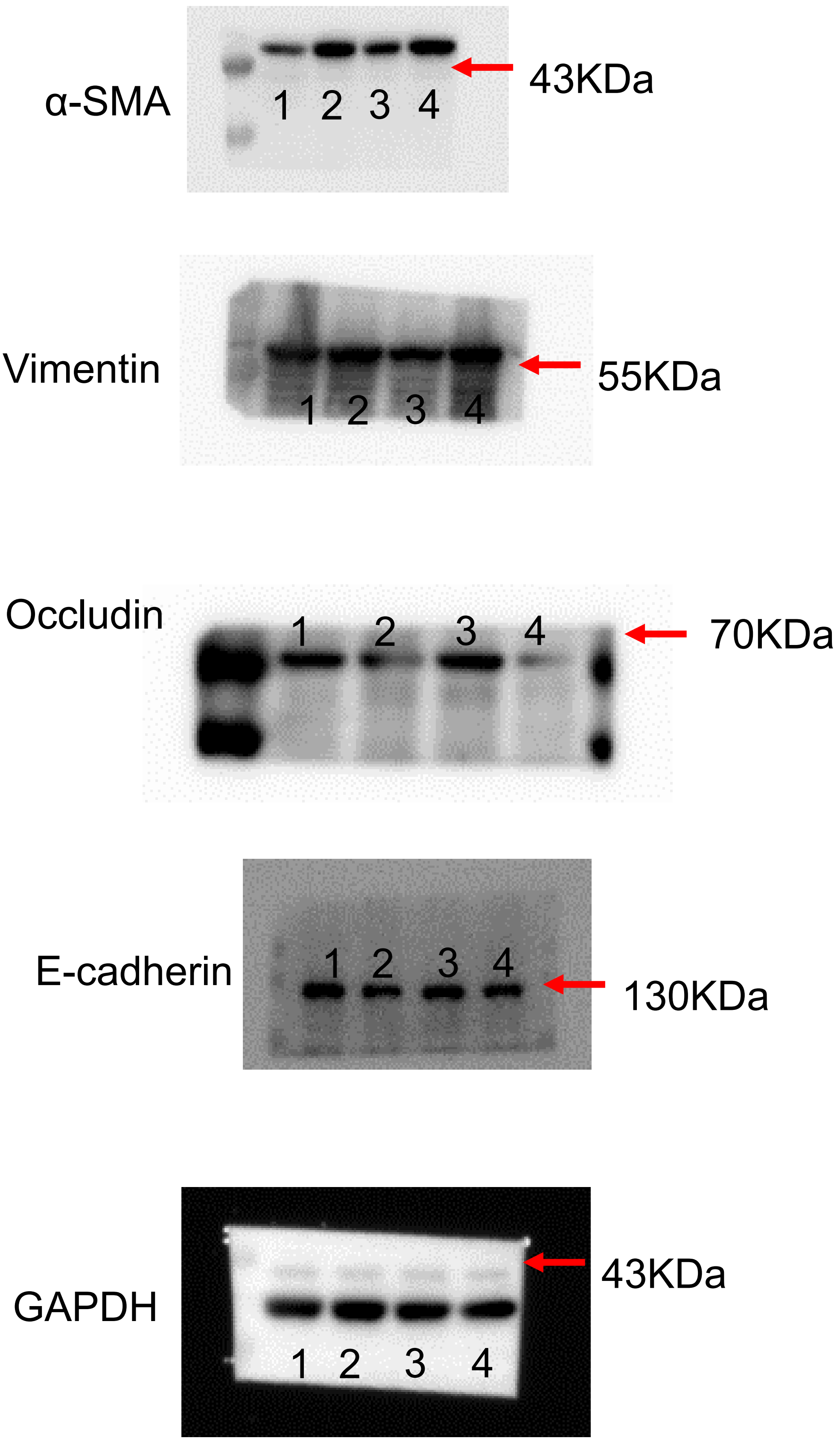

1： Control-50%

2： Control-25%

3： OE-CRX-25%

4： shCRX-25%

Supplementary figure.7J

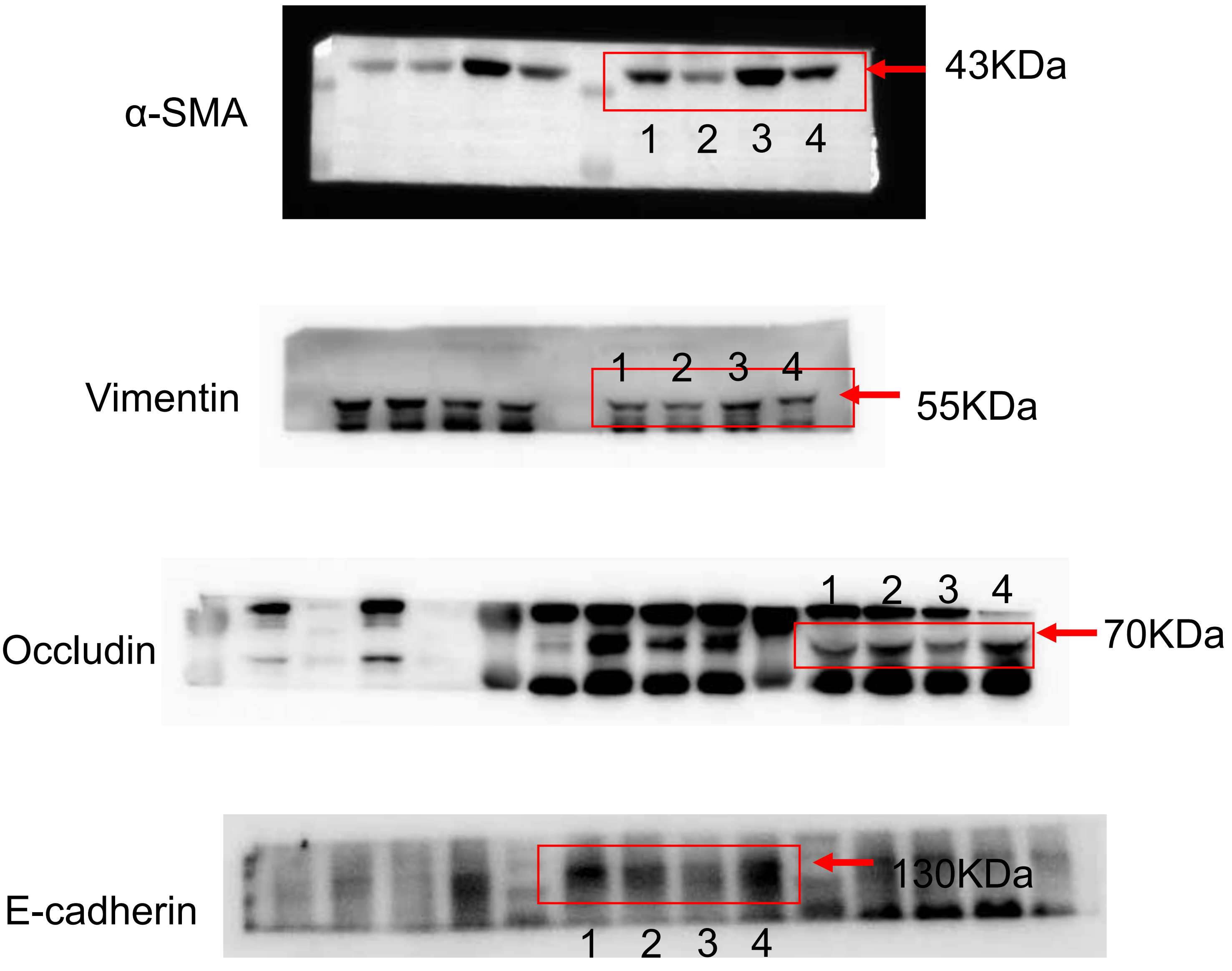

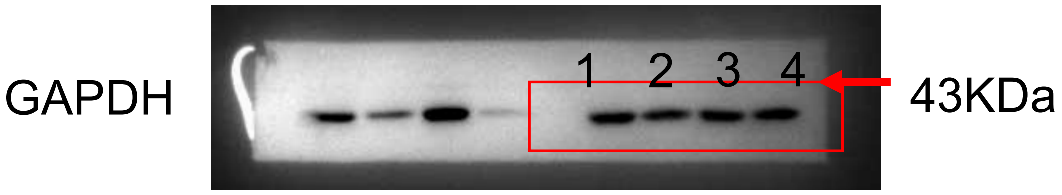

1： Control

2： OE-CRX

3： Control-TGF-β1

4： OE-CRX-TGF-β1
